# NF-kappaB p65-Dependent Transactivation of miRNA Genes following *Cryptosporidium parvum* Infection Stimulates Epithelial Cell Immune Responses

**DOI:** 10.1371/journal.ppat.1000681

**Published:** 2009-12-04

**Authors:** Rui Zhou, Guoku Hu, Jun Liu, Ai-Yu Gong, Kristen M. Drescher, Xian-Ming Chen

**Affiliations:** Department of Medical Microbiology and Immunology, Creighton University Medical Center, Omaha, Nebraska, United States of America; Albert Einstein College of Medicine, United States of America

## Abstract

*Cryptosporidium parvum* is a protozoan parasite that infects the gastrointestinal epithelium and causes diarrheal disease worldwide. Innate epithelial immune responses are key mediators of the host's defense to *C. parvum*. MicroRNAs (miRNAs) regulate gene expression at the posttranscriptional level and are involved in regulation of both innate and adaptive immune responses. Using an *in vitro* model of human cryptosporidiosis, we analyzed *C. parvum*-induced miRNA expression in biliary epithelial cells (i.e., cholangiocytes). Our results demonstrated differential alterations in the mature miRNA expression profile in cholangiocytes following *C. parvum* infection or lipopolysaccharide stimulation. Database analysis of *C. parvum*-upregulated miRNAs revealed potential NF-κB binding sites in the promoter elements of a subset of miRNA genes. We demonstrated that *mir-125b-1*, *mir-21*, *mir-30b*, *and mir-23b-27b-24-1* cluster genes were transactivated through promoter binding of the NF-κB p65 subunit following *C. parvum* infection. In contrast, *C. parvum* transactivated *mir-30c* and *mir-16* genes in cholangiocytes in a p65-independent manner. Importantly, functional inhibition of selected p65-dependent miRNAs in cholangiocytes increased *C. parvum* burden. Thus, we have identified a panel of miRNAs regulated through promoter binding of the NF-κB p65 subunit in human cholangiocytes in response to *C. parvum* infection, a process that may be relevant to the regulation of epithelial anti-microbial defense in general.

## Author Summary

MicroRNAs (miRNAs) are newly identified small non-coding RNAs that regulate gene expression at the posttranscriptional level. While much of our understanding of the cellular processes modulated by miRNAs has come from studies on development and tumorigenesis, the role of miRNAs in immune responses is now being gradually uncovered. Nevertheless, whether miRNA-mediated posttranscriptional gene regulation is involved in the fine-tuning of epithelial cell immune responses against pathogen infection remains undefined. *Cryptosporidium parvum* is a protozoan parasite that infects gastrointestinal epithelium. TLR/NF-κB-mediated innate immune responses by epithelial cells are critical to the host's defense to infection. Using an *in vitro* model of human cryptosporidiosis, we show here differential alterations in the miRNA expression profile in biliary epithelial cells following *C. parvum* infection. Promoter binding of NF-κB p65 subunit accounts for the upregulation of a panel of miRNA genes in cells infected by *C. parvum*. Importantly, functional inhibition of several NF-κB p65-dependent miRNAs in epithelial cells increases *C. parvum* infection burden. Our findings suggest that host epithelial cells activate NF-κB signaling to regulate miRNA expression in response to *C. parvum* infection. Moreover, NF-κB-mediated miRNA expression is involved in epithelial anti-microbial defense. Our study provides new insights into epithelial cell immunoregulation.

## Introduction

The protozoan parasite, *Cryptosporidium parvum*, is a causative agent of human gastrointestinal disease worldwide [1]. *C. parvum* infects the gastrointestinal epithelium to produce a self-limiting diarrhea in immunocompetent individuals but is potentially life-threatening in immunocompromised persons, especially those with the acquired immunodeficiency syndrome (AIDS) [1],[2]. Transmission occurs via the fecal-oral route. Humans are infected by ingesting *C. parvum* oocysts; oocysts then excyst in the gastrointestinal tract releasing infective sporozoites. *C. parvum* sporozoites can also travel up the biliary tract to infect the epithelial cells lining the biliary tract (i.e. cholangiocytes) [1],[3]. Mediated by specific ligands on the sporozoite surface and receptors on the host cells, the sporozoite attaches to the apical membrane of epithelial cells and forms a parasitophorous vacuole in which the organism remains intracellular but extracytoplasmic [3]. The sporozoite then matures and undergoes further development of its life cycle. With this unique extracytoplasmic niche within epithelial cells preventing a direct infection of other cell types, *C. parvum* is classified as a “minimally invasive” mucosal pathogen [1].

Because of the “minimally invasive” nature of *C. parvum* infection, innate immune responses by epithelial cells are critical to the host's defense against infection. Toll-like receptor (TLR) - and nuclear factor-kappaB (NF-κB) -mediated signaling pathways are important components in epithelial innate immunity to *C. parvum* infection [4],[5]. TLRs are transmembrane proteins with highly conserved structural domains [6]. Upon engagement of the TLRs by specific ligands, various adaptor molecules including myeloid differentiation factor 88 (MyD88) are selectively recruited to the receptors forming a complex referred to as the “signalosome” [6],[7]. The signalosome then triggers a series of downstream events including activation of the NF-κB [6]–[8]. NF-κB subunits bind to the κB sites within the promoters/enhancers of target genes resulting in the transcriptional regulation of multiple genes important to epithelial anti-*C. parvum* defense [4],[5].

MicroRNAs (miRNAs), a newly identified class of endogenous small regulatory RNAs of ∼24 nucleotides, are emerging as key mediators of many biological processes and impact gene expression at the posttranscriptional level [9],[10]. Similar to other RNA molecules, most of miRNAs are initially transcribed as primary transcripts (termed pri-miRNAs) by Poly II and processed by the RNase III Drosha (in the nucleus) and a second RNase III Dicer (in the cytoplasm) to generate mature miRNA molecules [11]–[13]. However, molecular mechanisms underlying miRNA gene transcriptional regulation are largely unclear [14]. Recent studies on expression of miRNA genes have revealed potential transcriptional regulation by transcription factors, such as NF-κB and C/EBPα [15],[16].

While much of our understanding of the cellular processes modulated by miRNAs has come from studies on development and tumorigenesis, the role of miRNAs in immune responses is now being gradually uncovered [17]–[19]. The importance of miRNAs in cell-mediated immunity is highlighted by Dicer conditional knockout mice. Specific deletion of *dcr-1* in the T cell lineage resulted in impaired T cell development and aberrant T helper cell differentiation and cytokine production [20]. In addition, miRNA expression is impacted by cytokines in some model systems. Both interferon (IFN) -α and IFN-β modulate expression of several miRNAs required for their anti-viral responses following infection with hepatitis C virus [21]. The TLR4 ligand, lipopolysaccharide (LPS), impacts expression of miR-132, miR-146, and miR-155 in human THP-1 monocytes [15],[22]. Further characterization of miR-146 revealed that this miRNA may function as a negative regulator of tumor necrosis factor receptor-associated factor 6 and interleukin-1 receptor associated kinase 1 [15]. Recent studies also implicate specific miRNAs in controlling various epithelial cell processes such as regulation of cellular differentiation, determination of epithelial cell fate (cell death and proliferation), initiation and regulation of anti-microbial immunity, fine-tuning of inflammatory responses, and activation of specific intracellular signaling pathways [17]–[19],[23]. Using a non-malignant human cholangiocyte cell line (H69) that expresses multiple TLRs including TLR4 [5], we previously demonstrated that infection of human cholangiocytes by *C. parvum in vitro* mimics parasitial apical invasion and TLR4/NF-κB-dependent epithelial responses *in vivo* [3]. Moreover, *let-7* regulates TLR4 expression via translational suppression in human cholangiocytes and is involved in epithelial defenses against *C. parvum* [24]. Members of the miR-98/*let-7* family also regulate expression of cytokine-inducible SH2-containing protein (CIS) in cholangiocytes following *C. parvum* infection [25]. Together, these findings demonstrate that miRNAs levels in epithelial cells are altered by *C. parvum* infection and may regulate epithelial anti-*C. parvum* immune responses.

In this study, we performed an array analysis of miRNA expression in H69 cells following *C. parvum* infection and LPS stimulation. The analysis revealed significant alterations in miRNA expression in cholangiocytes following *C. parvum* infection or treatment with LPS. Of those miRNAs upregulated by *C. parvum* infection, we identified potential NF-κB binding sites in the promoter elements of several miRNA genes. Inhibiting activation of NF-κB p65 blocked *C. parvum*-induced upregulation of a panel of miRNA genes. Promoter binding and transactivation of the NF-κB p65 subunit of each selected miRNA gene was confirmed by chromatin immunoprecipitation assay and promoter luciferase reporter analysis. Furthermore, functional inhibition of the NF-κB p65-binding miRNAs increased *C. parvum* burden in cholangiocytes *in vitro*. These data demonstrate that a panel of miRNAs is regulated through promoter binding of the NF-κB p65 subunit in human cholangiocytes and these miRNAs are involved in epithelial defense in response to *C. parvum* infection, suggesting a role of miRNAs in regulation of epithelial anti-microbial defense.

## Results

### *C. parvum* infection induces alterations in miRNA expression in cholangiocytes *in vitro*

**Figure 1:**
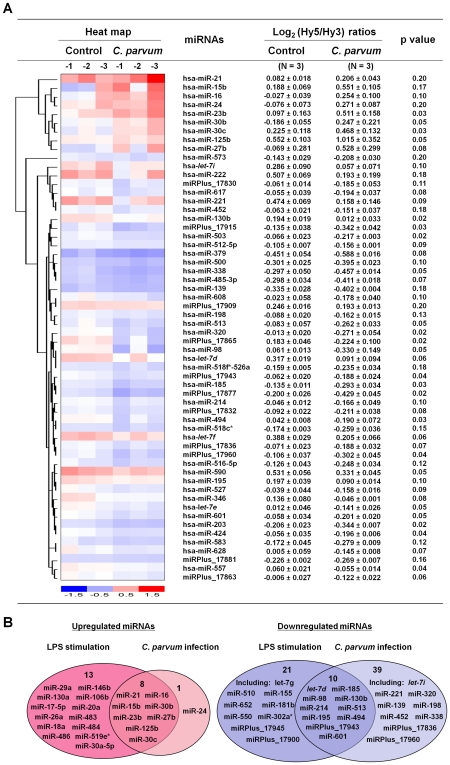
Expression profiling of mature miRNAs in cholangiocytes following *C. parvum* infection and LPS stimulation. (A) miRNA expression profile in H69 cells following *C. parvum* infection. The left panel shows a heat-map of selected miRNAs that showed changes in expression in H69 cells following *C. parvum* infection. The horizontal axis indicates samples of non-infected cells (n = 3; Control-1, -2, and -3) and cells after exposure to live *C. parvum* for 12 h (n = 3, *C. parvum*-1, -2, and -3). The right panel shows expression of miRNAs in H69 cells following *C. parvum* infection. Cellular levels of miRNAs were presented as the log_2_ (Hy5/Hy3) ratios which passed the filtering criteria variation across the samples. *p* values are from the t' test. hsa = *Homo sapiens*. (B) Comparison of miRNA expression patterns in H69 cells following *C. parvum* infection for 12 h and LPS stimulation for 8 h. Graphics indicate those miRNAs showing an increased or decreased expression (including those significant change when p< = 0.05 and those with a tendency to change when 0.05<p< = 0.20) in cells after treatment with LPS (n = 3) or exposure to *C. parvum* (n = 3). A complete description of miRNA expression profiles in cells was listed in Table S1.

To globally assess miRNA expression in epithelial cells following *C. parvum* infection, we performed a microarray analysis of mature miRNA expression in H69 cells [26]. The miRCURY™ LNA human microRNAs assays (version 8.1; Exiqon; Vedbaek, Denmark) covers a total of up to 600 known human mature miRNAs and were used as previously described [27]. The quality of the RNA was verified using an Agilent 2100 Bioanalyzer (Figure S1). A total of 383 mature miRNAs were detected in the uninfected H69 cells. Of the miRNAs expressed, miR-23b, miR-30b, miR-30c, and miR-125b expression were significantly increased in H69 cells after exposure to live *C. parvum* infection for 12 h (p< = 0.05; Figure 1A and Table S1). Five additional miRNAs (miR-15b, miR-16, miR-27b, miR-24, and miR-21) showed a tendency to increase (0.05<p< = 0.20) (Figure 1A). A total of 19 miRNAs were significantly downregulated (p< = 0.05) and 30 additional miRNAs showed a tendency to decrease (0.05<p< = 0.20) following *C. parvum* infection (Figure 1A and Table S1). Sham-infected control cells (H69 cells exposed to heat-inactivated *C. parvum* oocysts after incubation at 65°C for 30 min) displayed a similar miRNA expression profile as non-infected control samples (Table S1). Microarray analysis of mature miRNAs was also performed on H69 cells treated with LPS (1 µg/ml for 8 h). Interestingly, most of the miRNAs upregulated by *C. parvum* also displayed an increased expression in cells treated by LPS (Figure 1B and Table S1). Nevertheless, increased expression of additional 13 miRNAs was identified in LPS-treated cells but not in cells exposed to *C. parvum*. A total of 31 miRNAs showed a decreased expression in LPS-treated cells and 10 of them were also downregulated by *C. parvum* (Figure 1B and Table S1). No LPS contamination in the *C. parvum* preparation was detected using the Limulus Amebocyte Lysate (LAL) test kit (Bio-Whittaker) (data not shown). All microarray data were described in accordance with MIAME guidelines and deposited at ArrayExpress (accession number: E-MEXP-2050 and E-MEXP-2052).

**Figure 2:**
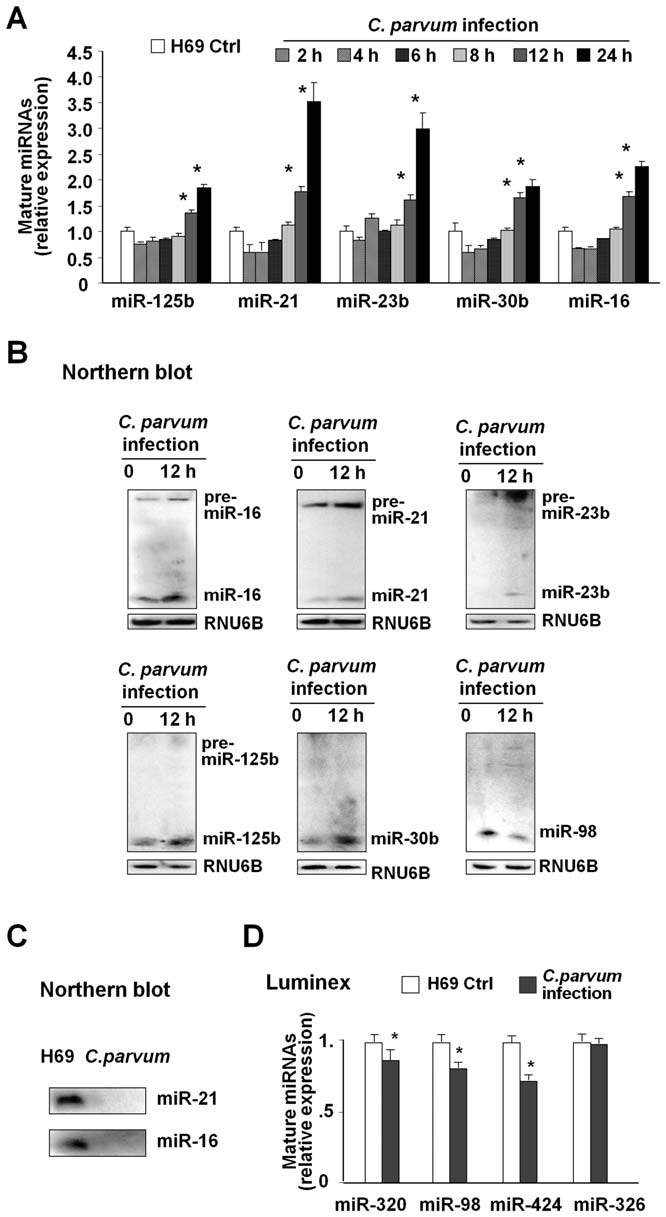
Altered expression of selected miRNAs confirmed by real-time PCR, Northern blot and Luminex bead analyses. (A) Alterations of selected miRNA expression in cells after exposure to *C. parvum* for various periods of time as assessed by real-time PCR. The amount of mature miRNAs was obtained by normalizing to the level of snRNA RNU6B in the samples. Data are expressed as the amount of mature miRNAs in the infected samples relative to the control uninfected samples and representative of three independent experiments. (B) Alterations of selected miRNA expression in cells after exposure to *C. parvum* for 12 h as determined by Northern blot. snRNA RNU6B was used as a control to ensure equal loading. Representative Northern blots (*C. parvum* infected cells vs non-infected control) from three independent experiments are shown. (C) Total RNA isolated from *C. parvum* oocysts was also blotted to demonstrate the specificity of the probes. (D) Alterations of selected miRNA expression in cells after exposure to *C. parvum* for 12 h as assessed by bead-based miRNA Luminex analysis. The amount of mature miRNAs was obtained by normalizing the samples to the positive control beads provided by the company (Luminex). Data are representative of three independent experiments. *, p<0.05 vs. the non-infected control.

Real-time PCR analysis using primers and probes for mature miRNAs (Ambion) was performed to assess the kinetics of selected miRNAs in H69 cells following *C. parvum* infection. Increased expression of miR-125b, miR-21, miR-23b, miR-30b and miR-16 was detected in H69 cells following *C. parvum* infection for 12 h to 24 h, but not in the early time points (2 h to 8 h) (Figure 2A). Increased expression of miR-125b, miR-16, miR-23b, miR-21 and miR-30b, as well as decreased expression of miR-98, was further confirmed in cells following *C. parvum* infection for 12 h by Northern blot (Figure 2B). An increased expression of the precursors for miR-125b, miR-16, miR-21 and miR-23b was also detected in cells following *C. parvum* infection by Northern blot (Figure 2B). No positive signal for the above human miRNAs was detected in *C. parvum* RNA using the probes or primers for miRNA real-time PCR (data not shown) and Northern blot (Figure 2C), demonstrating the specificity of these probes for human miRNAs. Downregulation of selected miRNAs induced by *C. parvum*, including miR-98, miR-320 and miR-424, was further confirmed by bead-based multiplexed miRNA expression assay using the FlexmiR™ Select kit (Figure 2D). For those miRNAs that did not show significant alterations in cells following *C. parvum* infection as revealed by the microarray analysis, we selected miR-326 for bead-based multiplexed analysis and no change was detected in *C. parvum* infected cells (Figure 2D), further confirming the accuracy of the array data.

### Database analysis of upregulated miRNAs in cholangiocytes following *C. parvum* infection reveals potential NF-κB binding sites in their promoter elements

Differential alterations in the mature miRNA expression profile of *C. parvum*-infected H69 cells suggest that miRNA gene expression is finely controlled in epithelial cells in response to *C. parvum* infection. One potential mechanism for selectively altering miRNA levels is through activation of distinct intracellular signaling pathways and nuclear transcription factors [15],[16]. This mechanism is consistent with our previous data demonstrating that *C. parvum* infection activates the NF-κB pathway in cholangiocytes through microbial recognition of TLR4 and TLR2 [28]. We hypothesized that activation of the NF-κB pathway is involved in the transcription of select miRNAs upregulated by *C. parvum*. Based on TFSEARCH (http://www.cbrc.jp/research/db/TFSEARCH.html) and MOTIF (http://motif.genome.jp/) database searches [29],[30], many of these miRNA genes have putative NF-κB binding sites in their potential promoter elements [31]–[34] (Table 1). Several miRNAs upregulated in H69 following *C. parvum* infection are cluster miRNAs; e.g., miR-23b, miR-27b and miR-24 are from the *mir-23b-27b-24-1* gene cluster and miR-15b and miR-16 from the *mir-15b-16-2* cluster [31],[32]. The promoters of the *mir-125b-1* and *mir-30b* genes have not been characterized and it is unknown whether they have potential NF-κB binding sites. Transactivation of most NF-κB-dependent genes requires NF-κB p65 binding to the promoter [8] and nuclear translocation of p65 was demonstrated following *C. parvum* infection of cholangiocytes [28]. Coupled with the results showing some similar changes in miRNA expression in H69 cells treated with LPS (which activates TLR4/NF-κB signaling in H69 cells), we then focused on determining whether p65 binds to the promoter and transactivates the miRNA genes upregulated by *C. parvum* infection.

**Table 1:** Analysis of *C. parvum*-upregulated miRNAs in cholangiocytes reveals potential transactivation of their genes by NF-κB.

Mature miRNAs	miRNA genes (or cluster)	Chromosome (strand)	Host gene	Predicted NF-kB binding sites (from miRNA TSS)	Reference
miR-125b	*mir-125b-1*	11 (−)	None	Promoter element unknown	[31]
	*mir-125b-2*	21 (+)	*C21orf34*	GAGAATTTCC (−893 to −884)	
miR-21	*mir-21*	17 (+)	None	GGGAATTTTC (+1167 to +1176)	[33],[34]
				GGGAATTCTC (+1395 to +1404)	
miR-23b	*mir-23b-27b-24-1*	9 (+)	*C9orf3*	GGGACTCTCC (−1263 to −1254)	[31]
miR-27b					
miR-24					
miR-30b	*mir-30b*	8 (−)	None	Promoter element unknown	
miR-30c	*mir-30c-1*	1 (+)	*NFYC*	TGGAATTACC (−689 to −680)	[31],[32]
	*mir-30c-2*	6 (−)	*C6orf155*	TGGGCTTTCC (−208 to −199)	
miR-16	*mir-15a-16-1*	13 (−)	*DLEU2*	None	[32]
miR-15a					
miR-16	*mir-15b-16-2*	3 (+)	*SMC4*	GGGATTTACC (−504 to −495)	[32]
miR-15b					

MicroRNA genes related to *C. parvum*-upregulated mature miRNAs, their chromosomal location and co-expressed host genes were identified by the miRBase (http://microrna.sanger.ac.uk/) database search and confirmed by previous studies as referred. Potential promoter element for each miRNA was based on the referred studies and potential NF-κB binding sites were identified by the TFSEARCH (http://www.cbrc.jp/research/db/TFSEARCH.html) and MOTIF (http://motif.genome.jp/) search.

### Differential expression of primary transcripts of *C. parvum*-upregulated mature miRNAs in H69 cells

**Figure 3:**
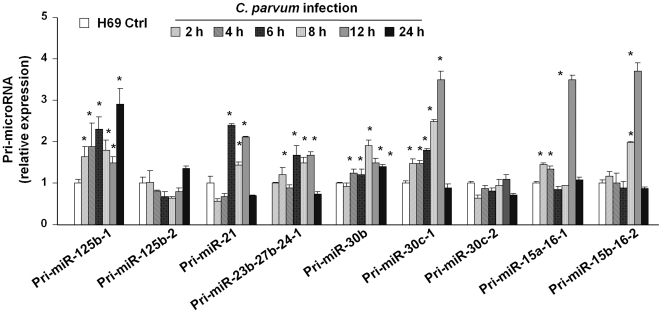
Differential expression of primary transcripts of *C. parvum*-upregulated mature miRNAs in H69 cells. H69 cells were exposed to *C. parvum* for 2 h to 24 h and primary transcripts (pri-miRNAs) of select miRNAs were quantified by real-time PCR. The amount of pri-miRNAs was obtained by normalizing to the level of GAPDH in the samples. Data are expressed as the amount of pri-miRNAs in the infected samples relative to the control uninfected samples and representative of three independent experiments. *, p<0.05 vs. the non-infected control.

We then analyzed the kinetics of alterations of the primary transcripts (pri-miRNAs) for select mature miRNAs upregulated by *C. parvum* as listed in Table 1. H69 cells were exposed to *C. parvum* for various periods of time and pri-miRNAs of interests were quantified by real-time PCR (primers listed in Table S2). Expression of pri-miR-125b-1, pri-miR-21, pri-miR-23b-27b-24-1, pri-miR-30b, pri-miR-30c-1, pri-miR-15a-16-1, and pri-miR-15b-16-2 showed a time-dependent increase in cells following *C. parvum* infection, with a peak at 8 h or 12 h after exposure to the parasite (Figure 3). In contrast, no significant increase of pri-miR-125b-2 and pri-miR-30c-2 was detected in cells after exposure to *C. parvum* infection (Figure 3), suggesting a differential expression of the primary transcripts of *C. parvum*-upregulated miRNAs.

### Promoter binding of NF-κB p65 subunit is required for the transcription of select miRNA genes induced by *C. parvum* in H69 cells

**Figure 4:**
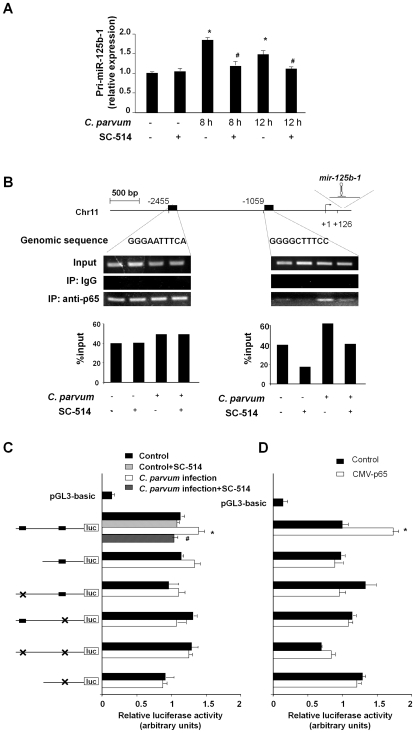
Promoter binding of p65 transactivates *miR-125b-1* gene to increase miR-125b expression following *C. parvum* infection. (A) p65-dependent upregulation of pri-miR-125b-1 in cholangiocytes following *C. parvum* infection. Data are presented as the relative expression level of pri-miR-125b-1 in H69 cells following *C. parvum* infection in the presence or absence of SC-514 as assessed by real-time PCR. (B) *C. parvum* increases promoter element binding of p65 to *mir-125b-1* gene. The schematic diagram shows two potential binding sites in the putative promoter element of *mir-125b-1* gene. ChIP analysis revealed increased binding of p65 to the binding site at −1059, but not at −2455, of *mir-125b-1* promoter element in cells following infection. Representative ChIP gels are shown in the upper panel and densitometry analysis of the gels in the lower panel. (C) H69 cells were transfected with various luciferase reporter constructs spanning the potential p65 binding sites of the *mir-125b-1* promoter. The transfected cells were exposed to *C. parvum* in the presence or absence of SC-514. Luciferase activity was measured and presented as the ratio of the activity of the test construct with the control luciferase reporter construct. Six reporter constructs containing the mutants of the two potential NF-κB binding sites were also utilized for the analysis as indicated. (D) H69 cells were co-transfected with the pCMV-p65 to overexpress p65 and the luciferase reporter construct containing the *mir-125b-1* promoter for 24 h followed by measurement of luciferase activity. *, p<0.05 vs. the non-infected control (in A and C) or empty pCMV vector control (in D); ^#^, p<0.05 vs. *C. parvum* infected cells (in A and C).

To test whether NF-κB p65 subunit is involved in *C. parvum*-induced transactivation of pri-miR-125b-1, we exposed H69 cells to *C. parvum* infection in the presence of SC-514, an IKK2 inhibitor that prevents p65-associated transcriptional activation of the NF-κB pathway [35]. SC-514 blocked the *C. parvum*-induced increase of pri-miR-125b-1 (Figure 4A). To further test the potential transactivation of *mir-125b-1* gene by p65 subunit, rapid amplification of 5′ complementary DNA ends (5′-RACE) PCR was used to identify the 5′ end of pri-miR-125b-1. Primers were designed to amplify pri-miR-125b-1 based on the sequence obtained from the Sanger miRNA Registry (Table S2). Database analysis revealed two potential p65 binding sites in the upstream sequence of *mir-125b-1* (Figure 4B). Increased binding of p65 to the binding site at −1059, but not the putative binding site at −2455, in the promoter element of *mir-125b-1* gene (Figure 4B) was demonstrated by chromatin immunoprecipitation (ChIP) analysis using specific primers for each putative binding site (Table S2). *C. parvum*-induced transactivation of the *mir-125b-1* gene by p65 was further confirmed by using luciferase reporter gene constructs that spanned the *mir-125b-1* promoter (Figure 4C). *C. parvum* infection increased luciferase activity in cells transfected with the luciferase constructs that encompassed the binding site for p65 at −1059 in the promoter region of *mir-125b-1* gene. A mutant of the p65 binding site at −1059 blocked *C. parvum*-induced luciferase activity. In addition, SC-514 significantly inhibited the increase of luciferase activity induced by *C. parvum* infection (Figure 4C). Moreover, p65-associated transactivation of the *mir-125b-1* promoter was also confirmed by the upregulation of luciferase activity after p65 overexpression in the cells (Figure 4D). As an additional control, we analyzed *IL-8* transactivation, a p65-dependent process induced by *C. parvum* in epithelial cells [36]. NF-κB p65-dependent increase of IL-8 mRNA expression and binding of p65 to the promoter of *IL-8* gene in cells exposed to *C. parvum* were confirmed (Figure S2). Together, these data demonstrate that p65 binding to the promoter element of the *mir-125b-1* gene mediates *mir-125b* upregulation in H69 cells in response to *C. parvum* infection. The dynamics of p65 nuclear translocation were confirmed by Western blot analysis of p65 in the nuclear extracts from H69 cells following *C. parvum* infection (Protocol S1 and Figure S3), correlated to the kinetics of *C. parvum*-induced expression of pri-miRNAs in cells (Figure 3). Consistent with previous results, maximal p65 translocation was observed at 8 h after exposure to *C. parvum* [5].

Using the same approaches, we analyzed p65 promoter element binding in *C. parvum*-induced transcription of pri-miR-21, pri-miR-23b-27b-24-1, pri-miR-30b, pri-miR-30c-1, pri-miR-30c-2, pri-miR-15a-16-1, and pri-miR-15b-16-2. Our data are summarized in Table 2 and presented in detail in Figures S4, S5, S6 and S7. Specifically, p65 binding to the putative p65 binding site around +1395 of the *mir-21* gene appears to be associated with *C. parvum*-induced transcription of pri-miR-21 (Figure S4). *C. parvum* increases transcription of pri-miR-23b-27b-24-1 cluster, as well as the host gene transcript, C9orf3, via promoter binding of p65 to a binding site at −1254 of the immediate upstream of the gene (Figure S5). Increased transcription of pri-miR-30b induced by *C. parvum* is p65-dependent (Figure S6). Nevertheless, it appears that *C. parvum* infection increases transcription of pri-miR-30c-1, pri-miR-15a-16-1 and pri-miR-15b-16-2 in cholangiocytes through a p65-independent mechanism (Figure S7).

**Table 2:** Promoter binding of NF-κB p65 subunit in *C. parvum*-induced transactivation of miRNA genes in H69 cells.

*C. parvum*-upregulated mature miRNAs	Corresponding pri-miRNAs	Uregulation of pri-miRNA by *C. parvum*	Inhibition of upregulation by SC-514	Potential NF-kB binding site(s) within the promoter region	Promoter p65 binding by ChIP	Conformed by promoter reporter assay
miR-125b	pri-miR-125b-1	+	+	GGGGCTTTCC(−1059 to −1050)*	+	+
				GGGAATTTCA (−2455 to −2446)*	−	−
	pri-miR-125b-2	−	−	GAGAATTTCC (−893 to −884)	NS	NS
miR-21	pri-miR-21	+	+	GGGAATTTTC (+1167 to +1176)	−	−
				GGGAATTCTC (+1395 to +1404)	+	+
miR-23b	pri-miR-23b-27b-24-1	+	+	GGGACTCTCC (−1263 to −1254)	+	+
miR-27b						
miR-24-1						
miR-30b	pri-miR-30b	+	+	AGGAATTTAC (−347 to −338)*	+	+
miR-30c	pri-miR-30c-1	+	−	TGGAATTACC (−689 to −680)	NS	NS
	pri-miR-30c-2	−	−	TGGGCTTTCC (−208 to −199)	NS	NS
miR-16	pri-miR-15a-16-1	+	−	None	NS	NS
miR-15a						
miR-16	pri-miR-15b-16-2	+	−	GGGATTTACC (−504 to −495)	NS	NS
miR-15b						

Expression of pri-miRNAs corresponding to *C. parvum*-upregulated mature miRNAs was quantified by real-time PCR. These showed significant upregulation in cells following *C. parvum* infection were presented as “+” and those without significant increase were presented as “−”. Effects of NF-κB inhibitor SC-514 on *C. parvum*-induced upregulation of pri-miRNAs were also assessed by real-time PCR and presented as “+” (if the inhibitory effect was significant) and “−” (if not significant). Putative promoter element for each miRNA gene was either based on previous studies as referred in Table 1 or identified by RACE-PCR in this study (indicated by asterisks). Potential NF-κB binding site(s) in the promoter region was identified by the TFSEARCH (http://www.cbrc.jp/research/db/TFSEARCH.html) and MOTIF (http://motif.genome.jp/). Positive promoter binding of p65 subunit to the predicted NF-κB binding site was confirmed by ChIP analysis and marked as “+”; otherwise marked as “−” if no p65 binding was detected. *C. parvum*-induced transactivation of miRNA gene by p65 was further confirmed by using luciferase reporter gene constructs that spanned the promoter region of each individual gene. If *C. parvum* infection increased luciferase activity in cells transfected with the luciferase constructs containing the binding site for p65 and this induction was blocked by SC-514, it was presented as “+”; otherwise presented as “−”. NS = not selected for further ChIP analysis or luciferase reporter assay in this study. In such cases, these genes are most likely not regulated by p65 promoter binding because their transactivation was not induced by *C. parvum* infection or the induced transactivation was not inhibited by SC-514 as assessed by real-time PCR. Refer to Figures S4, S5, S6 and S7 for details.

### Functional inhibition of selected p65-dependent miRNAs in cholangiocytes increases *C. parvum* infection burden

**Figure 5:**
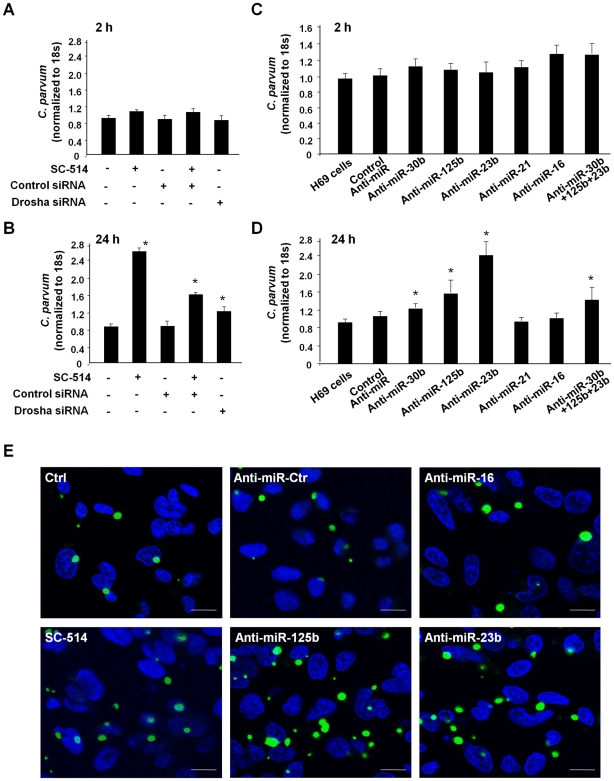
Functional inhibition of selected p65-dependent miRNAs in cholangiocytes increases *C. parvum* infection burden *in vitro*. (A) A similar number of parasites was detected in cells transfected with Drosha siRNA or treated with SC-514 after initial exposure to *C. parvum* for 2 h as quantified by real-time PCR. (B) Transfection of cells with Drosha siRNA or treated with SC-514 increased *C. parvum* infection burden in cholangiocytes *in vitro* 24 h after initial exposure to the parasite. (C) Effects of anti-miRs on *C. parvum* after initial exposure to *C. parvum* for 2 h. (D) Transfection of cells with anti-miRs on *C. parvum* infection burden in cholangiocytes 24 h after initial exposure to the parasite. *, *p*<0.05 vs. non-treated cells or cells transfected with a control siRNA (in B) or non-specific control anti-miR (in D). (E) Effects of anti-miRs or SC-514 on *C. parvum* burden in cholangiocytes *in vitro* 24 h after initial exposure to the parasite as assessed by immunofluorescent microscopy. *C. parvum* parasites were stained in green and nuclei in blue. Bars = 5 µm.

To test whether miRNAs are involved in cholangiocyte defense responses against *C. parvum* infection, we assessed parasite burden over time in cultured cholangiocytes transfected with various anti-miRs thereby inhibiting function of specific *C. parvum*-upregulated miRNAs. Anti-miRs (anti-miR™ miRNA inhibitors) are commercially available, chemically modified single stranded nucleic acids designed to specifically bind to and inhibit endogenous miRNAs [21]. Cells were transfected with specific anti-miRs (30 nM, Ambion) or a mixture of anti-miRs to miR-125b, miR-23b and miR-30b (a total of 30 nM with 10 nM for each), and then exposed to *C. parvum*. Following incubation with a constant number of *C. parvum* sporozoites for 2 h to allow sufficient host-cell attachment and cellular invasion [3],[24], cells were washed with culture medium to remove non-attached and non-internalized parasites. Cells were then cultured for an additional 2 h or 22 h. Parasite burden was assessed in the samples using a real-time PCR approach as we previously reported [24]. The parasite burden following exposure to *C. parvum* for 2 h was similar in all cultures, including those transfected with the siRNA to Drosha or the specific anti-miRs (Figure 5A and 5C), suggesting that those miRNAs do not affect initial parasite host cell attachment and cellular invasion. Additionally, SC-514 treatment did not impact parasite burden at this time point (Figure 5A). Consistent with our previous studies [24], a significant increase in parasite burden was identified in SC-514-treated H69 cells 24 h after initial infection (Figure 5B). Cells transfected with the siRNA to Drosha displayed an increased parasite burden as compared to control cells (Figure 5B). Interestingly, we also detected a significantly higher parasite burden 24 h after initial infection in cells treated with the anti-miRs to miR-125b, miR-23b, and miR-30b, as well as a mixture of three anti-miRs, compared with that in control cells (Figure 5D); anti-miRs to miR-16 and miR-21 did not impact infection burden (Figure 5D). Increase of parasite burden 24 h after initial infection in H69 cells treated with SC-514 or select anti-miRs was further confirmed by immunofluorescent microscopy (Figure 5E).

The targets of a majority of known miRNAs are still yet to be identified. *C. parvum*-responsive miRNAs may regulate the expression of proteins of various functions related to epithelial anti-*C. parvum* defense. Using computational analyses as previously reported [19], [37]–[39], we identified a variety of potential targets of *C. parvum*-responsive miRNAs selected on the basis of their known involvement in immune related responses (Table S3).

## Discussion

There is emerging evidence that miRNAs play a critical role in the regulation of both innate and adaptive immunity [17]–[19]. A better understanding miRNA expression changes in epithelial cells following *C. parvum* infection will provide new insights in miRNA-associated epithelial defense to *C. parvum*. Using an *in vitro* model of human biliary cryptosporidiosis, we report significant alterations in miRNA expression profiles in epithelial cells following *C. parvum* infection. Our analysis of miRNAs upregulated by *C. parvum* in H69 cells revealed that *mir-125b-1*, *mir-23b-27b-24-1*, *mir-21*, and *mir-30b* genes are transactivated via potential promoter binding of the NF-κB p65 subunit. These data provide several insights relevant to miRNA expression regulation in cholangiocytes following *C. parvum* infection. First, similar to the regulation of miRNA genes in other cells [16],[40],[41], promoter binding of transcription factors regulates miRNA genes in epithelial cells in response to *C. parvum* infection. Therefore, transcription factor-mediated miRNA expression and subsequent gene regulation at the posttranscriptional level through miRNA targeting may be an important element of host responses against *C. parvum* infection. Since similar alterations in miRNA expression profile were identified in LPS-treated cells, this observation may also be relevant to cellular gene regulation in general. Second, transactivation of miRNA genes that produce the same mature miRNA can be differentially controlled. Specifically, both *mir-125b-1* and *mir-125b-2* genes can produce mature miR-125b, but only transactivation of *mir-125b-1* gene was detected in cells following *C. parvum* infection. Indeed, differential activation of genes for the same mature miRNA molecule has been previously reported [31]. Finally, transactivation of genes of cluster miRNAs or as introns in other gene alleles may be controlled by the same promoter element. Of note, miR-23b, miR-27b and miR-24 are cluster gene miRNAs and co-transcribed with a host gene, *C9orf3* [31]. *C. parvum* infection upregulates expression of the mature forms of these three miRNAs, as well as pri-miR-23b-27b-24-1 and the host gene transcript, C9orf3. Our data are consistent with recent studies demonstrating transcriptional control of genes that code cluster miRNAs or that encode both miRNAs and other host transcripts [31],[32].

The NF-κB family of transcription factors consists of five members, p50, p52, p65 (RelA), c-Rel, and RelB [8]. The transcription activation domain (TAD) necessary for the positive regulation of gene expression is present only in p65, c-Rel, and RelB [8]. Thus, promoter binding of p65, c-Rel and RelB is usually associated with gene transactivation [8], [42]–[44]. Because they lack TADs, p50 and p52 may repress transcription unless they associate with a TAD-containing NF-κB family member or another protein capable of coactivator recruitment [8],[45],[46]. Increased nuclear translocation of p65 and p50 was previously reported in human cholangiocytes following *C. parvum* infection [28]. In this study, we demonstrated that promoter binding of the NF-κB p65 subunit is required for transactivation of the *mir-125b-1*, *mir-23b-27b-24-1*, *mir-21* and *mir-30b* genes in cells following *C. parvum* infection. Although transactivation of *mir-30c-1* and *mir-15b-16-2* genes was observed in *C. parvum*-infected cells and potential NF-κB binding sites were identified in their promoter elements, inhibition of p65 activation failed to inhibit transactivation of either *mir-30c-1* or *mir-15b-16-2* in H69 cells following *C. parvum*-infection. In addition, miR-146b, miR-155, and miR-9 have been reported to be NF-κB-dependent miRNAs in monocytes or neutrophils [15],[47],[48]. Although miR-146b and miR-155 are expressed in cholangiocytes, no upregulation of either miR-146b or miR-155 was detected in H69 cells following *C. parvum* infection. Given the complexity and variability in the gene structure for each miRNA, it is obvious that multiple mechanisms are involved in the transcriptional regulation of human miRNA genes [32],[33],[49]. Therefore, transcription of miRNA genes is expected to be a dynamic process in response to the constant alterations in intracellular signals. miRNA expression thus reflects the final integrated result of multiple interrelated signals on miRNA transcription. In this regard, other transcription factors, such as AP-1, c-myc, C/EBPα, may also be involved in the transcriptional regulation of miRNA genes in epithelial cells in response to *C. parvum* infection. Future studies will focus on whether nuclear translocation of p50 is involved in the *C. parvum*-induced down-regulation of miRNA expression.

miRNAs have been identified in both mammalian and nonmammalian cells including virus and parasites [9],[14],[50],[51]. Expression of miRNAs in *C. parvum* has not yet been examined and whether *C. parvum*-derived miRNAs can be localized in infected host cells is unknown. Nevertheless, the probes used in the microarray analysis in this study are human-miRNA specific with minimal cross-interaction with known miRNAs from other species. Cells of sham-infection control (host cells plus heat-inactivated *C. parvum* oocysts) displayed a similar expression profile of human miRNAs compared with non-infected control cells as assessed by microarray analysis. Finally, by Northern blot and real-time PCR, no positive signal was detected in *C. parvum* RNA alone using the probes/primers for selected human miRNAs, confirming host-cell specificity of detected miRNAs.

The TLR/NF-κB signaling is critical to innate epithelial immune defenses to microbial infection including parasites [4],[52]. We previously demonstrated that TLR4 and TLR2 are involved in cholangiocyte immune response to *C. parvum* infection via activation of NF-κB [5]. Here, we expanded our previous studies by demonstrating that miRNAs may also regulate TLR/NF-κB-mediated epithelial anti-*C. parvum* defense. We indentified a panel of miRNA genes that are transactivated via p65 promoter binding in cholangiocytes in response to *C. parvum* infection. Transfection of cells with anti-miRs to miR-125b, miR-23b or miR-30b, but not anti-miRs to miR-16 or miR-21, significantly increased parasite burden in cholangiocytes. The molecular mechanisms by which *C. parvum*-responsive miRNAs modulate epithelial anti-*C. parvum* defense are largely unclear. Previous studies demonstrated that *let-7* regulates TLR4 expression and is involved in epithelial defense against *C. parvum* [24]. Various immune related genes are identified as potential targets for these *C. parvum*-responsive miRNAs using computational analyses. The concept that a pathogen encodes mRNAs targeted by host miRNAs has recently emerged as an important mechanism of host anti-viral defense [21]. Likewise, it is of interest to test the possibility that host cell miRNAs target the internalized parasite mRNAs and silence genes of the pathogen. The direct *C. parvum*-host cell cytoplasmic tunnel-connection [53] could mediate exchange of molecules, including miRNAs, between the host cells and internalized parasite. Further investigation should test whether p65 promoter binding transactivates LPS-responsive miRNA genes. This also raises the possibility that transactivation of miRNA genes through promoter binding of NF-κB subunits may be involved in host anti-microbial responses in general.

In summary, this first miRNA profiling in cholangiocytes in response to *C. parvum* infection *in vitro* revealed significant alterations in cellular miRNA expression. The mechanism by which *C. parvum* induces upregulation of a panel of miRNAs in cholangiocytes involves transactivation of miRNA genes through promoter binding of the NF-κB p65 subunit. In addition, functional inhibition of the upregulated miRNAs increases *C. parvum* infection burden in cholangiocytes *in vitro* thereby implicating these miRNAs in host cell defense to the parasite. These data demonstrate a key role for miRNAs in epithelial immune responses against *C. parvum* infection and may provide new insights into general mechanisms of the regulation of epithelial anti-microbial immunity.

## Materials and Methods

### *C. parvum* and human cholangiocyte cell line

*C. parvum* oocysts of the Iowa strain were purchased from a commercial source (Bunch Grass Farm, Deary, ID). H69 cells (a gift of Dr. D. Jefferson, Tufts University) are SV40 transformed normal human cholangiocytes originally derived from liver harvested for transplant. These cholangiocytes continue to express biliary epithelial cell markers, including cytokeratin 19, gamma glutamyl transpeptidase and ion transporters consistent with biliary function and have been extensively characterized [26].

### *In vitro* infection model and infection assay

An *in vitro* model of human biliary cryptosporidiosis using H69 cells was employed in these studies. Before infecting cells, *C. parvum* oocysts were treated with 1% sodium hypochlorite on ice for 20 min followed by extensive washing with DMEM-F12 medium. Oocysts were then added to the cell culture to release sporozoites to infect cells [54]. Infection was performed in culture medium (DMEM-F12 with 100 U/ml penicillin and 100 µg/ml streptomycin) containing viable *C. parvum* oocysts (oocysts with host cells in a 5∶1 ratio). Inactivated organisms (treated at 65°C for 30 min) were used for sham infection controls. All experiments were performed in triplicate. For the inhibition experiments, SC-514 (Calbiochem) was added to the medium. Cells were pre-treated with SC-514 for 1 h prior to *C. parvum* infection. SC-514 was used at a concentration of 100 µM, which showed no cytotoxic effects on H69 cells or on *C. parvum* sporozoites, in these studies.

Real-time PCR and immunofluorescent microscopy were used to assay *C. parvum* infection as previously reported [24]. Briefly, primers specific for *C. parvum* 18s ribosomal RNA (forward: 5′-TAGAGATTGGAGGTTGTTCCT-3′ and reverse: 5′-CTCCACCAACTAAGAACGGCC-3′) were used to amplify the cDNA specific to the parasite. Primers specific for human plus *C. parvum* 18s were used to determine total 18s cDNA [24]. Data were expressed as copies of *C. parvum* 18s vs total 18s. For immunofluorescent microscopy, cells were fixed with 2% paraformaldehyde and incubated with a polyclonal antibody against *C. parvum* (a gift from Dr. Guan Zhu, Texas A&M University) followed by anti-rabbit FITC-conjugated secondary antibody (Molecular Probes) and co-staining with 4′, 6-diamidino-2-phenylindole (DAPI, 5 µM) to stain cell nuclei. Labeled cells were assessed by confocal laser scanning microscopy.

### miRCURY™ LNA array analysis of miRNAs

The Exiqon (Vedbaek, Denmark) miRCURY LNA microRNA arrays and service to process the samples were used [27]. Briefly, H69 cells were grown to 80% confluence and exposed to *C. parvum* oocysts for 12 h or LPS (1 µg/ml) for 8 h. Total RNAs from H69 cells or *C. parvum* oocysts were prepared with the mirVana™ miRNA Isolation Kit according to the manufacturer's instruction (Ambion). The quality of isolated RNAs was verified by an Agilent 2100 Bioanalyzer profile (Figure S1). A mixture of equal amounts of total RNAs from the control and *C. parvum*-infected cells were used as the reference pool. A total of 2 µg RNA from each sample was then labeled with the Hy5™ fluorescent label and the reference pool labeled with Hy3™ using the miRCURY™ LNA Array labeling kit (Exiqon). The labeled samples and reference pool were then mixed pair-wise and hybridized to the miRCURY™ LNA array containing capture probes targeting all human miRNAs listed in the miRBASE version 8.1 (Exiqon). After hybridization, the slides were scanned and quantified signals normalized by Exiqon using the global Lowess (Locally Weighted Scatterplot Smoothing) regression algorithm. Normalized Hy5/Hy3 ratios were used for further analysis as previously reported [55]–[57].

### Bead-based multiplex sandwich immunoassays

A bead-based multiplex sandwich immunoassay, read with a Luminex 200 system (Luminex), was used to measure the concentrations of selected miRNAs as previously reported [57]. Briefly, total cellular RNAs are isolated using the mirVana™ miRNA Isolation Kit (Ambion). An amount of 0.5 µg of total RNAs was used for Biotin-labeling using the FlexmiR MicroRNA Labeling Kit for selected miRNAs (Luminex). Signals for miRNAs were recorded and standardized to the standard beads according to the manufacturer's instructions (Luminex).

### Real-time PCR

For real-time PCR analysis of mature miRNAs, total RNAs were extracted using the mirVana™ miRNA Isolation kit (Ambion). An amount of 0.05 µg total RNAs was reverse-transcribed by using the Taqman MicroRNA Reverse Transcription Kit (Applied Biosystems). Comparative real-time PCR was performed in triplicate using Taqman Universal PCR Master Mix (Applied Biosystems) on the Applied Biosystems 7500 FAST real-time PCR System. Mature miRNA-specific primers and probes were obtained from Applied Biosystems. Normalization was performed by using RNU6B primers and probes. Relative expression was calculated by using the comparative CT method [56].

For analysis of pri-miRNAs, total RNA was isolated from cells with Trizol reagent (Ambion). RNAs were treated with DNA-free™ Kit (Ambion) to remove any remaining DNA. Comparative real-time PCR was performed by using the SYBR Green PCR Master Mix (Applied Biosystems). Specific primers for pri-miRNAs were listed in Table S2. All reactions were run in triplicate. The Ct values were analyzed using the comparative Ct (ΔΔCt) method and the amount of target was obtained by normalizing to the endogenous reference (GAPDH) and relative to the control (non-treated cells) [58].

### Northern blot

Total RNAs harvested as above were run on a 15% Tris/Borate/EDTA (90 mM Tris/64.6 mM boric acid/2.5 mM EDTA, pH 8.3)–urea gel (Invitrogen) and transferred to a Nytran nylon transfer membrane (Ambion). LNA DIG-probes for selected miRNAs (Exiqon) were hybridized using UltraHyb reagents (Ambion) according to the manufacturer's instructions with blotted snRNA RNU6B as a control.

### 5′-RACE PCR

5′-RACE PCR was utilized to identify 5′ end of miRNA primary transcripts to localize the start sites of *mir-125b-1*, *mir-30b* and *mir-30d*. Primer sequences are listed in Table S2. The SMART™ RACE cDNA Amplification Kit (Clontech) was used for the analysis. Total RNA was isolated for H69 cells treated with a Drosha siRNA (Santa Cruz biotechnology) as previously reported [32].

### ChIP

ChIP analysis was performed with a commercially available ChIP Assay Kit (Upstate Biotechnologies) in accordance with the manufacturer's instructions. In brief, 1×10^6^ H69 cells were cultured in 15-cm culture dishes and exposed to *C. parvum* in the presence or absence of SC-514 for 8 h. The chromatin fraction was immunoprecipitated for overnight at 4°C using anti-NF-κB p65 (Upstate Biotechnologies). PCR amplification was performed in a total volume of 25 µl with specific primers. The forward and reverse primers used for each gene were listed in Table S2.

### Luciferase reporter constructs and luciferase assay

Promoters of miRNAs were amplified by PCR from human genomic DNA. PCR primers were listed in Table S2. The PCR products were separated by agarose gel electrophoresis, and the DNA fragments then isolated and cloned in the restriction enzyme digested pGL3 Basic Vector (Promega) using T4 DNA ligase (Fisher scientific). All constructs were confirmed by sequencing. Mutations were introduced into the NF-κB binding sites using the QuikChange site-directed mutagenesis kit (Stratagene). H69 cells were transfected with each reporter construct for 24 h and then exposed to *C. parvum* oocysts for 8 h in the presence or absence of SC-514 followed by assessment of luciferase activity. Luciferase activities were then measured and normalized to the control β-gal level. The luciferase activity of each construct was compared with that of the promoterless pGL3 basic vector.

## Supporting Information

Table S1 miRNA expression profile in cholangiocytes following *C. parvum* infection and LPS stimulation. Data represent the mean±SE of the log_2_ (Hy5/Hy3) ratios from non-infected cell cultures (n = 3), *C. parvum* infected cultures (n = 3), LPS treated cultures (n = 3), and one cell culture exposed to heated-inactivated *C. parvum* (Sham) by using the miRCURY™ LNA Array (Version 8.1). ^a^, p< = 0.05; ^b^, 0.05<p< = 0.20, compared with non-infected cells; NA = not detectable.
(0.09 MB PDF)

Table S2 Primers used for PCR and construct generating. Listed in this table are all the primers used in this study for the real-time PCR and RACE PCR, as well as those for ChIP analysis and construct generating. ^a^Restriction enzyme sites were indicated by lowercase letters.
(0.02 MB PDF)

Table S3 Prediction of immune-related target genes of *C. parvum*-responsive miRNAs. Prediction of immune-related target genes for *C. parvum*-responsive miRNAs was performed with the computerlized predictive algorithms as previously reported [19], [37]–[39]. Some of the predicted targets have been experimentally confirmed [24],[25],[27],[59],[60] and the corresponding miRNAs are in red font.
(0.02 MB PDF)

Figure S1 Quality control of RNA. Total RNAs from cells were prepared with the mirVana™ miRNA Isolation Kit according to the manufacturer's instructions (Ambion). The quality of the isolated RNAs was verified by examining the Agilent 2100 Bioanalyzer profile of the sample. Representative RNA profiles from non-infected H69 cells (A), cells exposed to live (B) and heat-inactivated *C. parvum* oocysts (C) are shown.
(1.62 MB TIF)

Figure S2 Promoter binding of p65 transactivates the *IL-8* gene in cholangiocytes in response to *C. parvum* infection. (A) p65-dependent upregulation of IL-8 mRNA in cholangiocytes following *C. parvum* infection. Bars represent the levels of IL-8 mRNA in cells following *C. parvum* infection in the presence or absence of SC-514 as assessed by real-time PCR. (B) A schematic diagram shows the structure of *IL-8* gene. ChIP analysis demonstrated increased binding of p65 to the binding site at *IL-8* promoter in cells following infection. *, p<0.05 vs. non-infected cells; #, p<0.05 vs. *C. parvum* infected cells.
(0.28 MB TIF)

Figure S3 Nuclear translocation of p65 in cholangiocytes cells induced by *C. parvum*. Cells were exposed to *C. parvum* for various periods of time and nuclear extracts obtained as described in Protocol S1. The NF-κB p65 subunit was detected by Western blot. Actin was used as a loading control. Representative Western blots are shown.
(0.61 MB TIF)

Figure S4 Promoter binding of p65 transactivates *mir-21* gene to increase miR-21 expression in biliary epithelial cells in response to *C. parvum* infection. (A) p65-dependent upregulation of pri-miR-21 in cholangiocytes following *C. parvum* infection. A schematic diagram illustrates the structure of the *mir-21* gene that encodes human miR-21. Bars represent the expression levels of pri-miR-21 in cells following *C. parvum* infection in the presence or absence of SC-514 as assessed by real-time PCR. (B) *C. parvum* increases promoter binding of p65 to the *mir-21 gene*. ChIP analysis revealed increased binding of p65 to the promoter binding site at +1395, but not at +1167 in H69 cells following infection. (C) H69 cells were transfected with various luciferase reporter constructs covering the potential binding sites of the *mir-21* promoter and then exposed to *C. parvum* in the presence or absence of SC-514. A mutant at +1395 blocked *C. parvum*-induced luciferase reporter activity in transfected cells. (D) H69 cells were co-transfected with the pCMV-p65 to overexpress p65 and the luciferase reporter construct containing the *mir-21* promoter. Different from the results in *C. parvum*-infected cells, a significant increase of luciferase reporter activity was detected in cells co-transfected with the pCMV-p65 and the mutant at +1167. *, p<0.05 vs. the non-infected control (in A and C) or empty pCMV vector control (in D); #, p<0.05 vs. *C. parvum* infected cells (in A and C).
(0.63 MB TIF)

Figure S5 Promoter binding of p65 transactivates the *mir-23b-27b-24-1* cluster gene in cholangiocytes in response to *C. parvum* infection. (A) p65-dependent upregulation of pri-miR-23b-27b-24-1 in cholangiocytes following *C. parvum* infection. A schematic diagram shows the structure of the *mir-23b-27b-24-1* cluster gene. Real-time PCR was used to assess the expression levels of pri-miRNA-23b-27b-24-1 and C9orf3 following *C. parvum* infection in the presence or absence of SC-514. (B) *C. parvum* increases promoter binding of p65 to the *mir-23b-27b-24-1* cluster gene. The schematic diagram shows one potential NF-κB binding site in the promoter element of *mir-23b-27b-24-1*. ChIP analysis revealed increased binding of p65 to the binding site at −1254 of the promoter in cells following infection. (C) H69 cells were transfected with luciferase gene reporter constructs with or without mutations in the p65 binding site of the promoter and then exposed to *C. parvum* in the presence or absence of SC-514. (D) H69 cells were co-transfected with the pCMV-p65 to overexpress p65 and the luciferase reporter gene construct containing the promoter. Cells were then cultured for 24 h followed by measurement of luciferase activity. *, p<0.05 vs. the non-infected control (in A and C) or empty pCMV vector control (in D); #, p<0.05 vs. *C. parvum* infected cells (in A and C).
(0.76 MB TIF)

Figure S6 Promoter binding of p65 transactivates the *mir-30b* gene to increase miR-30b expression in biliary epithelial cells following *C. parvum* infection. (A) p65-dependent upregulation of pri-miR-30b, but not pri-miR-30d, in cholangiocytes following *C. parvum* infection. The expression levels of pri-miRNAs in cells following *C. parvum* infection were assessed by real time PCR in the presence or absence of SC-514. Treatment of cells with SC-514 blocked *C. parvum*-induced increase of pri-miR-30b, suggesting p65-dependent miR-30b expression. (B) We performed 5′-RACE PCR to identify the 5′end of pri-miR-30d and identified a potential p65 binding site at −472 of its upstream sequence. H69 cells were transfected with the luciferase gene reporter construct covering the potential p65 binding site within the putative promoter of mir-30d and then exposed to *C. parvum*. These results support that pri-miR-30b and pri-miR-30d are not transcribed from the same gene in human cholangiocytes, inconsistent with previous results suggesting that pri-miR-30b and pri-miR-30d may be transcribed from the same gene on chr8 [31],[40]. (C) To clarify how p65 is involved in the transactivation of miR-30b gene transactivation, we performed 5′-RACE PCR but failed to amplify the corresponding sequence (data not shown). Nevertheless, database analysis revealed one potential binding site for NF-κB in the upstream sequence of miR-30b precursor. ChIP analysis detected an increased binding of p65 to this region in cells following *C. parvum* infection. (D) Luciferase reporter gene analysis demonstrated a significant increase in luciferase reporter activity in cells following *C. parvum* infection or overexpressed with p65. *, p<0.05 vs. the non-infected control (in A and D) or empty pCMV vector control (in E); #, p<0.05 vs. *C. parvum* infected cells (in A and D).
(0.73 MB TIF)

Figure S7 p65-independent expression of miR-30c and miR-16 in cholangiocytes in response to *C. parvum* infection. (A and B) miR-30c is transcribed from two genes, *mir-30c-1* and *mir-30c-2*, localized on chr1 and chr6, respectively [31]. Real-time PCR analysis revealed an increase of pri-miR-30c-1(A), but not pri-miR-30c-2 (B), in H69 cells following *C. parvum* infection. Treatment of cells with SC-514 failed to block *C. parvum*-induced expression of pri-miR-30c-1 (A). (C and D) miR-16 is transcribed from two genes, *mir-15a-16-1* and *mir-15b-16-2* localized on chr13 and chr3 and clustered with miR-15a and miR-15b, respectively [31]. Increased expression of pri-miR-15a-16-1 (at 12 h; C) and pri-miR-15b-16-2 (at 8 h and 12 h; D) was detected in H69 cells after *C. parvum* infection. Treatment of cells with SC-514 failed to block either pri-miR-15a-16-1 (C) or pri-miR-15b-16-2 (D). *, p<0.05 vs. the non-infected control.
(0.58 MB TIF)

Protocol S1 Nuclear translocation of p65.
(0.01 MB PDF)

## References

[1] Chen XM, Keithly JS, Paya CV, LaRusso NF (2002). Cryptosporidiosis.. N Engl J Med.

[2] Wanyiri J, Ward H (2006). Molecular basis of *Cryptosporidium*-host cell interactions: recent advances and future prospects.. Future Microbiol.

[3] Chen XM, Levine SA, Tietz P, Krueger E, LaRusso NF (1998). *Cryptosporidium parvum* is cytopathic for cultured human biliary epithelia via an apoptotic mechanism.. Hepatology.

[4] Rogers KA, Rogers AB, Leav BA, Sanchez A, Vannier E (2006). MyD88-dependent pathways mediate resistance to *Cryptosporidium parvum* infection in mice.. Infect Immun.

[5] Chen XM, Nelson JB, O'Hara SP, Splinter PL, Small AJ (2005). Multiple Toll-like Receptors are expressed in human cholangiocytes and mediate host epithelial responses to *Cryptoaporidium parvum* via activation of NF-kappaB.. J Immunol.

[6] Akira S, Takeda K (2004). Toll-like receptor signalling.. Nat Rev Immunol.

[7] Iwasaki A, Medzhitov R (2004). Toll-like receptor control of the adaptive immune responses.. Nat Immunol.

[8] Hayden MS, Ghosh S (2008). Shared principles in NF-κB signaling.. Cell.

[9] Bartel DP (2004). MicroRNAs: genomics, biogenesis, mechanism, and function.. Cell.

[10] Ambros V (2004). The functions of animal microRNAs.. Nature.

[11] Lee Y, Kim M, Han J, Yeom KH, Lee S (2004). MicroRNA genes are transcribed by RNA polymerase II.. EMBO J.

[12] Ozsolak F, Poling LL, Wang Z, Liu H, Liu XS (2008). Chromatin structure analyses identify miRNA promoters.. Genes Dev.

[13] Kim YK, Kim VN (2007). Processing of intronic microRNAs.. EMBO J.

[14] Winter J, Jung S, Keller S, Gregory RI, Diederichs S (2009). Many roads to maturity: microRNA biogenesis pathways and their regulation.. Nat Cell Biol.

[15] Taganov KD, Boldin MP, Chang KJ, Baltimore D (2006). NF-kappaB-dependent induction of microRNA miR-146, an inhibitor targeted to signaling proteins of innate immune responses.. Proc Natl Acad Sci U S A.

[16] Fazi F, Rosa A, Fatica A, Gelmetti V, De Marchis ML (2005). A minicircuitry comprised of microRNA-223 and transcription factors NFI-A and C/EBPalpha regulates human granulopoiesis.. Cell.

[17] Baltimore D, Boldin MP, O'Connell RM, Rao DS, Taganov KD (2008). MicroRNAs: new regulators of immune cell development and function.. Nat Immunol.

[18] Liu J, Drescher KM, Chen XM (2009). MicroRNAs and Epithelial Immunity.. Int Rev Immunol.

[19] Asirvatham AJ, Gregorie CJ, Hu Z, Magner WJ, Tomasi TB (2008). MicroRNA targets in immune genes and the Dicer/Argonaute and ARE machinery components.. Mol Immunol.

[20] Muljo SA, Ansel KM, Kanellopoulou C, Livingston DM, Rao A (2005). Aberrant T cell differentiation in the absence of Dicer.. J Exp Med.

[21] Pedersen IM, Cheng G, Wieland S, Volinia S, Croce CM (2007). Interferon modulation of cellular microRNAs as an antiviral mechanism.. Nature.

[22] O'Connell RM, Taganov KD, Boldin MP, Cheng G, Baltimore D (2007). MicroRNA-155 is induced during the macrophage inflammatory response.. Proc Natl Acad Sci U S A.

[23] Friedman RC, Farh KK, Burge CB, Bartel DP (2009). Most mammalian mRNAs are conserved targets of microRNAs.. Genome Research.

[24] Chen XM, Splinter PL, O'Hara SP, LaRusso NF (2007). A cellular miRNA, *let-7i*, regulates toll-like receptor 4 expression and contributes to cholangiocyte immune responses against *Cryptosporidium parvum* infection.. J Biol Chem.

[25] Hu G, Zhou R, Liu J, Gong A-Y, Eischeid A (2009). MicroRNA-98 and *let-7* confer cholangiocyte expression of cytokine-inducible Src homology 2-containing protein in response to microbial challenge.. J Immunol.

[26] Grubman SA, Perrone RD, Lee DW, Murray SL, Rogers LC (1994). Regulation of intracellular pH by immortalized human intrahepatic biliary epithelial cell lines.. Am J Physiol.

[27] Gong A-Y, Zhou R, Hu G, Li X, Splinter PL (2009). MicroRNA-513 regulates B7-H1 translation and is involved in interferon-gamma-induced B7-H1 expression in cholangiocytes.. J Immunol.

[28] Chen XM, Levine SA, Splinter PL, Tietz PS, Ganong AL (2001). *Cryptosporidium parvum* activates nuclear factor kappaB in biliary epithelia preventing epithelial cell apoptosis.. Gastroenterology.

[29] Kast C, Wang M, Whiteway M (2003). The ERK/MAPK pathway regulates the activity of the human tissue factor pathway inhibitor-2 promoter.. J Biol Chem.

[30] Musikacharoen T, Matsuguchi T, Kikuchi T, Yoshikai Y (2001). NF-kappa B and STAT5 play important roles in the regulation of mouse Toll-like receptor 2 gene expression.. J Immunol.

[31] Rodriguez A, Griffiths-Jones S, Ashurst JL, Bradley A (2004). Identification of mammalian microRNA host genes and transcription units.. Genome Res.

[32] Chang TC, Yu D, Lee YS, Wentzel EA, Arking DE (2008). Widespread microRNA repression by Myc contributes to tumorigenesis.. Nat Genet.

[33] Löffler D, Brocke-Heidrich K, Pfeifer G, Stocsits C, Hackermüller J (2007). Interleukin-6 dependent survival of multiple myeloma cells involves the Stat3-mediated induction of microRNA-21 through a highly conserved enhancer.. Blood.

[34] Cai X, Hagedorn CH, Cullen BR (2004). Human microRNAs are processed from capped, polyadenylated transcripts that can also function as mRNAs.. RNA.

[35] Kishore N, Sommers C, Mathialagan S, Guzova J, Yao M (2003). A selective IKK-2 inhibitor blocks NF-kappa B-dependent gene expression in interleukin-1 beta-stimulated synovial fibroblasts.. J Biol Chem.

[36] Laurent F, Eckmann L, Savidge TC, Morgan G, Theodos C (1997). *Cryptosporidium parvum* infection of human intestinal epithelial cells induces the polarized secretion of C-X-C chemokines.. Infect Immun.

[37] Schmidt WM, Spiel AO, Jilma B, Wüller M (2009). In vivo profile of the human leukocyte microRNA response to endotoxemia.. Biochem Biophys Res Commun.

[38] Grimson A, Farh KK, Johnston WK, Garrett-Engele P, Lim LP (2007). MicroRNA targeting specificity in mammals: determinants beyond seed pairing.. Mol Cell.

[39] Betel D, Wilson M, Gabow A, Marks DS, Sander C (2007). The microRNA.org resource: targets and expression.. Nucleic Acid Res.

[40] Marson A, Levine SS, Cole MF, Frampton GM, Brambrink T (2008). Connecting microRNA genes to the core transcriptional regulatory circuitry of embryonic stem cells.. Cell.

[41] Song G, Wang L (2008). Transcriptional mechanism for the paired miR-433 and miR-127 genes by nuclear receptors SHP and ERRc.. Nucleic Acid Res.

[42] Abreu MT, Fukata M, Arditi M (2005). TLR signaling in the gut in health and disease.. J Immunol.

[43] Han J, Ulevitch RJ (2005). Limiting inflammatory responses during activation of innate immunity.. Nat Immunol.

[44] Harada K, Ohira S, Isse K, Ozaki S, Zen Y (2003). Lipopolysaccharide activates nuclear factor-kappaB through toll-like receptors and related molecules in cultured biliary epithelial cells.. Lab Invest.

[45] Poppelmann B, Klimmek K, Strozyk E, Voss R, Schwarz T (2005). NF{kappa}B-dependent down-regulation of tumor necrosis factor receptor-associated proteins contributes to interleukin-1-mediated enhancement of ultraviolet B-induced apoptosis.. J Biol Chem.

[46] Kim S, Domon-Dell C, Kang J, Chung DH, Freund JN (2004). Down-regulation of the tumor suppressor PTEN by the tumor necrosis factor-alpha/nuclear factor-kappaB (NF-kappaB)-inducing kinase/NF-kappaB pathway is linked to a default IkappaB-alpha autoregulatory loop.. J Biol Chem.

[47] Bazzoni F, Rossato M, Fabbri M, Gaudiosi D, Mirolo M (2009). Induction and regulatory function of miR-9 in human monocytes and neutrophils exposed to proinflammatory signals.. Proc Natl Acad Sci U S A.

[48] Tili E, Michaille JJ, Cimino A, Costinean S, Dumitru CD (2007). Modulation of miR-155 and miR-125b levels following lipopolysaccharide/TNF-alpha stimulation and their possible roles in regulating the response to endotoxin shock.. J Immunol.

[49] Saini HK, Griffiths-Jones S, Enright AJ (2007). Genomic analysis of human microRNA transcripts.. Proc Natl Acad Sci U S A.

[50] Xue X, Sun J, Zhang Q, Wang Z, Huang Y (2008). Identification and characterization of novel microRNAs from Schistosoma japonicum.. PLoS ONE.

[51] Cerutti H, Casas-Mollano JA (2006). On the origin and functions of RNA-mediated silencing: from protists to man.. Curr Genet.

[52] Zaph C, Troy AE, Taylor BC, Berman-Booty LD, Guild KJ (2007). Epithelial-cell-intrinsic IKK-beta expression regulates intestinal immune homeostasis.. Nature.

[53] Huang BQ, Chen XM, LaRusso NF (2004). *Cryptosporidium parvum* attachment to and internalization by human biliary epithelia *in vitro*: a morphologic study.. J Parasitol.

[54] Verdon R, Keusch GT, Tzipor S, Grubman SA, Jefferson DM (1997). An *in vitro* model of infection of human biliary epithelial cells by *Cryptosporidium parvum*.. J Infect Di.

[55] Castoldi M, Schmidt S, Benes V, Noerholm M, Kulozik AE (2006). A sensitive array for microRNA expression profiling (miChip) based on locked nucleic acids (LNA).. RNA.

[56] Loscher CJ, Hokamp K, Kenna PF, Ivens AC, Humphries P (2007). Altered retinal microRNA expression profile in a mouse model of retinitis pigmentosa.. Genome Biol.

[57] Lu J, Getz G, Miska EA, Alvarez-Saavedra E, Lamb J (2005). MicroRNA expression profiles classify human cancers.. Nature.

[58] Davis BN, Hilyard AC, Lagna G, Hata A (2008). SMAD proteins control DROSHA-mediated microRNA maturation.. Nature.

[59] Cimmino A, Calin GA, Fabbri M, Iorio MV, Ferracin M (2005). miR-15 and miR-16 induce apoptosis by targeting BCL2.. Proc Natl Acad Sci U S A.

[60] Ueda R, Kohanbash G, Sasaki K, Fujita M, Zhu X (2009). Dicer-regulated microRNAs 222 and 339 promote resistance of cancer cells to cytotoxic T-lymphocytes by down-regulation of ICAM-1.. Proc Natl Acad Sci U S A.

