# Supplementary material for: NF-kappaB p65-Dependent Transactivation of miRNA Genes following Cryptosporidium parvum Infection Stimulates Epithelial Cell Immune Responses
Source: PLoS Pathog. 2009 Dec 4;5(12):e1000681. doi: 10.1371/journal.ppat.1000681 (PMC2778997; doi:10.1371/journal.ppat.1000681)
Supplement: Table S1 — miRNA expression profile in cholangiocytes following C. parvum infection and LPS stimulation. Data represent the mean±SE of the log2 (Hy5/Hy3) ratios from non-infected cell cultures (n = 3), C. parvum infected cultures (n = 3), LPS treated cultures (n = 3), and one cell culture exposed to heated-inactivated C. parvum (Sham) by using the miRCURY™ LNA Array (Version 8.1). a, p< = 0.05; b, 0.05<p< = 0.20, compared with non-infected cells; NA = not detectable. (0.09 MB PDF) [file ppat.1000681.s001.pdf]

Table S1. miRNA expression profile in cholangiocytes following *C. parvum* infection and LPS stimulation

miRNAs	Log$_2$(Hy5/Hy3) ratios			
	Control (n=3)	*C. parvum* (n=3)	LPS (n=3)	Sham (n=1)
hsa-*let-7d*	0.318 ±0.019	0.091 ±0.094[b]	-0.042 ±0.056[a]	0.599
hsa-*let-7f*	0.389 ±0.029	0.205 ±0.067[b]	NA	0.139
hsa-*let-7g*	0.454 ±0.009	0.309 ±0.071	0.060 ±0.018[a]	0.318
hsa-*let-7e*	0.013 ±0.047	-0.141 ±0.026[a]	-0.079 ±0.066	0.535
hsa-*let-7i*	0.286 ±0.090	0.058 ±0.072[b]	0.005 ±0.187	0.077
hsa-miR-106b	0.135 ±0.047	0.465 ±0.317	1.176 ±0.394[a]	0.738
hsa-miR-124a	-0.450 ±0.059	-0.601 ±0.069	-0.690 ±0.020[a]	-0.394
hsa-miR-125b	0.082 ±0.018	0.206 ±0.044[a]	0.332 ±0.036[a]	0.608
hsa-miR-130a	0.086 ±0.055	0.313 ±0.102	0.804 ±0.276[b]	0.260
hsa-miR-130b	0.195 ±0.019	0.012 ±0.033[a]	0.002 ±0.049[a]	0.330
hsa-miR-139	-0.335 ±0.028	-0.402 ±0.005[b]	-0.393 ±0.032	-0.222
hsa-miR-146b	-0.042 ±0.133	0.139 ±0.169	0.730 ±0.136[a]	0.389
hsa-miR-155	-0.178 ±0.025	-0.199 ±0.041	-0.404 ±0.038[a]	-0.094
hsa-miR-15b	-0.069 ±0.282	0.528 ±0.299[b]	0.924 ±0.560[b]	-0.045
hsa-miR-16	0.097 ±0.163	0.512 ±0.158[b]	1.086 ±0.353[b]	0.415
hsa-miR-17-5p	0.200 ±0.074	0.529 ±0.198	1.156 ±0.401[b]	0.454
hsa-miR-181b	-0.234 ±0.083	-0.305 ±0.019	-0.436 ±0.077	-0.229
hsa-miR-18a	0.207 ±0.033	0.396 ±0.161	0.863 ±0.194[a]	0.271
hsa-miR-185	-0.136 ±0.012	-0.294 ±0.034[a]	-0.338 ±0.113[b]	-0.274
hsa-miR-195	0.197 ±0.039	0.091 ±0.015[b]	0.076 ±0.014[a]	0.520
hsa-miR-198	-0.088 ±0.020	-0.162 ±0.016[b]	-0.109 ±0.038	-0.016
hsa-miR-20a	0.264 ±0.058	0.551 ±0.200	1.167 ±0.406[b]	0.597
hsa-miR-203	-0.207 ±0.023	-0.345 ±0.008[a]	-0.253 ±0.060	-0.077
hsa-miR-208	-0.145 ±0.022	-0.187 ±0.020	-0.359 ±0.021	-0.145
hsa-miR-21	0.553 ±0.104	1.015 ±0.352[b]	1.853 ±0.535[b]	0.802
hsa-miR-214	-0.046 ±0.012	-0.167 ±0.050[b]	-0.207 ±0.110[b]	-0.009
hsa-miR-221	0.475 ±0.070	0.159 ±0.147[b]	-0.069 ±0.257	0.194
hsa-miR-222	0.508 ±0.069	0.194 ±0.200[b]	0.410 ±0.247	0.086
hsa-miR-23b	0.189 ±0.070	0.552 ±0.105[a]	0.933 ±0.431[b]	0.332
hsa-miR-24	0.225 ±0.119	0.468 ±0.132[b]	0.716 ±0.371	0.583
hsa-miR-26a	-0.512 ±0.115	-0.193 ±0.116	0.340 ±0.256[a]	-0.472
hsa-miR-27b	-0.186 ±0.055	0.248 ±0.222[b]	0.743 ±0.595[b]	0.213
hsa-miR-29a	0.407 ±0.032	0.314 ±0.185	1.269 ±0.392[b]	0.284
hsa-miR-302a*	-0.305 ±0.027	-0.404 ±0.016	-0.554 ±0.041	-0.230
hsa-miR-30a-5p	-0.166 ±0.081	0.122 ±0.176	0.713 ±0.401[b]	0.434
hsa-miR-30c	-0.076 ±0.074	0.272 ±0.087[a]	0.610 ±0.296[b]	0.107
hsa-miR-30b	-0.028 ±0.039	0.254 ±0.100[a]	0.851 ±0.384[b]	0.226
hsa-miR-320	-0.013 ±0.020	-0.271 ±0.054[a]	-0.213 ±0.144	-0.219
hsa-miR-338	-0.297 ±0.050	-0.457 ±0.015[a]	-0.521 ±0.103	-0.271
hsa-miR-346	0.136 ±0.081	-0.046 ±0.002[b]	-0.085 ±0.140	0.178
hsa-miR-379	-0.452 ±0.054	-0.588 ±0.016[b]	-0.537 ±0.058	-0.295
hsa-miR-424	-0.057 ±0.036	-0.196 ±0.007[a]	-0.195 ±0.047	0.187
hsa-miR-452	-0.064 ±0.021	-0.152 ±0.038[b]	-0.083 ±0.039	0.065
hsa-miR-483	-1.377 ±0.092	-1.349 ±0.041	-0.882 ±0.090[a]	-1.510
hsa-miR-484	-0.863 ±0.059	-0.874 ±0.026	-0.467 ±0.024[a]	-0.921
hsa-miR-485-3p	-0.298 ±0.034	-0.411 ±0.018[b]	-0.418 ±0.040	-0.215
hsa-miR-486	-0.703 ±0.046	-0.759 ±0.032	-0.378 ±0.034a	-0.775
hsa-miR-490	0.064 ±0.039	0.015 ±0.069	-0.135 ±0.075[b]	0.634
hsa-miR-492	-0.041 ±0.046	-0.042 ±0.138	-0.228 ±0.077[b]	-0.443
hsa-miR-494	0.042 ±0.008	-0.190 ±0.073[a]	-0.345 ±0.266[b]	-0.231

hsa-miR-500	-0.301 ±0.026	-0.395 ±0.024[b]	-0.258 ±0.130	-0.221
hsa-miR-503	-0.066 ±0.023	-0.217 ±0.003[a]	-0.357 ±0.222	-0.134
hsa-miR-510	0.063 ±0.017	-0.071 ±0.048	-0.121 ±0.127[b]	0.326
hsa-miR-512-5p	-0.105 ±0.007	-0.156 ±0.002[b]	-0.146 ±0.026	-0.149
hsa-miR-513	-0.083 ±0.057	-0.262 ±0.033[a]	-0.601 ±0.458[b]	-0.236
hsa-miR-516-5p	-0.126 ±0.043	-0.248 ±0.035[b]	-0.204 ±0.092	0.320
hsa-miR-518c*	-0.174 ±0.003	-0.260 ±0.036[b]	-0.198 ±0.096	-0.232
hsa-miR-518f*-526a	-0.159 ±0.006	-0.235 ±0.035[b]	-0.171 ±0.047	-0.154
hsa-miR-519e*	-0.272 ±0.017	-0.258 ±0.016	-0.068 ±0.059[a]	-0.207
hsa-miR-520d*	0.074 ±0.039	-0.060 ±0.085	-0.313 ±0.095[a]	-0.057
hsa-miR-524*	0.187 ±0.050	0.117 ±0.051	-0.130 ±0.044[a]	0.082
hsa-miR-526b	0.106 ±0.067	0.001 ±0.044	-0.230 ±0.038[a]	0.165
hsa-miR-527	-0.040 ±0.045	-0.159 ±0.016[b]	-0.124 ±0.068	0.534
hsa-miR-550	0.359 ±0.054	0.337 ±0.027	0.101 ±0.068[a]	0.338
hsa-miR-557	0.060 ±0.022	-0.055 ±0.014[a]	-0.014 ±0.047	0.477
hsa-miR-573	-0.144 ±0.029	-0.208 ±0.030[b]	-0.104 ±0.084	-0.083
hsa-miR-583	-0.173 ±0.045	-0.280 ±0.009[b]	-0.275 ±0.148	-0.178
hsa-miR-584	0.068 ±0.044	-0.046 ±0.047	-0.115 ±0.035[a]	0.357
hsa-miR-588	-0.201 ±0.006	-0.184 ±0.022	-0.322 ±0.027[a]	-0.145
hsa-miR-590	0.532 ±0.056	0.331 ±0.045[a]	NA	0.199
hsa-miR-601	-0.059 ±0.034	-0.201 ±0.020[a]	-0.330 ±0.092[a]	0.382
hsa-miR-603	-0.224 ±0.053	-0.301 ±0.030	-0.420 ±0.006[a]	-0.137
hsa-miR-608	-0.023 ±0.058	-0.178 ±0.040[b]	-0.381 ±0.124	0.038
hsa-miR-617	-0.056 ±0.040	-0.194 ±0.037[b]	-0.213 ±0.087	0.079
hsa-miR-628	0.006 ±0.059	-0.145 ±0.008[b]	-0.190 ±0.079	0.492
hsa-miR-652	0.061 ±0.025	0.039 ±0.064	-0.095 ±0.048	0.026
hsa-miR-98	0.062 ±0.014	-0.330 ±0.149[a]	-0.403 ±0.157[a]	0.023
miRPlus_17830	-0.062 ±0.014	-0.186 ±0.053[b]	-0.178 ±0.058	0.090
miRPlus_17832	-0.093 ±0.022	-0.212 ±0.038[b]	-0.145 ±0.089	-0.058
miRPlus_17836	-0.072 ±0.023	-0.188 ±0.033[b]	-0.286 ±0.157	-0.091
miRPlus_17845	0.194 ±0.042	0.046 ±0.042	-0.105 ±0.065[a]	0.102
miRPlus_17861	-0.169 ±0.031	-0.238 ±0.042	-0.099 ±0.074	-0.107
miRPlus_17863	-0.007 ±0.027	-0.123 ±0.022[b]	-0.099 ±0.198	0.399
miRPlus_17865	0.184 ±0.046	-0.225 ±0.101[a]	0.083 ±0.217	-0.184
miRPlus_17870	0.373 ±0.012	0.328 ±0.021	0.184 ±0.040[a]	0.304
miRPlus_17877	-0.200 ±0.026	-0.429 ±0.046[a]	-0.390 ±0.292	-0.283
miRPlus_17881	-0.226 ±0.002	-0.270 ±0.008[b]	-0.317 ±0.233	-0.427
miRPlus_17900	0.351 ±0.057	0.268 ±0.051	0.176 ±0.029[a]	0.633
miRPlus_17909	0.247 ±0.017	0.193 ±0.014[b]	0.140 ±0.112	0.109
miRPlus_17915	-0.135 ±0.039	-0.342 ±0.042[a]	-0.159 ±0.242	-0.057
miRPlus_17943	-0.062 ±0.021	-0.188 ±0.024[a]	-0.271 ±0.120[b]	0.375
miRPlus_17960	-0.107 ±0.037	-0.303 ±0.045[a]	-0.337 ±0.309	-0.285

Data represent the mean ± SE of the $\log_2$ (Hy5/Hy3) ratios from non-infected cell cultures (n = 3), *C. parvum* infected cultures (n = 3), LPS treated cultures (n = 3), and one cell culture exposed to heated-inactivated *C. parvum* (Sham) by using the miRCURYTM LNA Array (Version 8.1). [a], p < = 0.05; [b], 0.05 < p <= 0.20; NA = not detectable.
