# Supplementary material for: NF-kappaB p65-Dependent Transactivation of miRNA Genes following Cryptosporidium parvum Infection Stimulates Epithelial Cell Immune Responses
Source: PLoS Pathog. 2009 Dec 4;5(12):e1000681. doi: 10.1371/journal.ppat.1000681 (PMC2778997; doi:10.1371/journal.ppat.1000681)
Supplement: Table S2 — Primers used for PCR and construct generating. Listed in this table are all the primers used in this study for the real-time PCR and RACE PCR, as well as those for ChIP analysis and construct generating. aRestriction enzyme sites were indicated by lowercase letters. (0.02 MB PDF) [file ppat.1000681.s002.pdf]

Table S2. Primers used for PCR and construct generating.

	Sense primer (5'-3')	Antisense primer (5'-3')
**Primers for real-time PCR**		
**Pri-miR-23b-24-1**	TCACATTGCCAGGGATTACCA	TGCACCTGTTCTCCAATCTGC
**Pri-miR-125b-1**	CCATACCACCTGTTTGTTGCATCT	CTGAGAGGAGCGCAACAATGT
**Pri-miR-125b-2**	GAAGAATTCTACCGCATCAAACCA	CTGCAGACAATCAATAAGGTCCAA
**Pri-miR-21**	TTTTGTTTTGCTTGGGAGGA	AGCAGACAGTCAGGCAGGAT
**Pri-miR-15a-16-1**	AAGGTGCAGGCCATATTGTG	AAGGCACTGCTGACATTGC
**Pri-miR-15b-16-2**	CATGCTACAGTCAAGATGCGAATC	CGTGCTGCTAGAGTGGAACAAGT
**Pri-miR-30c-1**	CTGTGGGCTATAACCATGCTGTAG	GATCTGCGGAGTGGAGACTGTT
**Pri-miR-30c-2**	CCTAGAGAGCACTGAGCGACAGA	TTCTCCCAGCTTTCTTACTTTCCA
**Pri-miR-30b**	TGGGAGGTGGATGTTTACTTCAG	GGTCTCACATTTCCAACAAACCTT
**Pri-miR-30d**	TTGCTGCTACCGGCTATTCAC	CAACTCCACTCCGGGACAGA
**C9orf3**	CAACGCAGCTTTGGGAAGAGT	GAGGCTGGCATTGGCATAGTT
**IL-8**	ATGACTTCCAAGCTGGCCGT	CCTCTTCAAAAACTTCTCCACACC
**GAPDH**	TGCACCACCAACTGCTTAGC	GGCATGGACTGTGGTCATGAG
**Primers for Chromatin Immunoprecipitation**		
mir-23b-27b-24-1	AGCAGCTAGCAGGGTGATGT	CATGGGAAGAACAGAGGATGA
***mir-125b-1* (-2455)**	CGTCCATAAAGAAAGGCCAC	CGCAAGACCTAGAGATCAGG
***mir-125b-1* (-1059)**	TCATCTTCCCATCTGCCT	CTGCGGATTCTTTGAAGC
***mir-21* (+1167)**	GGAGTGGATGGGTTCTGCCTTA	CAAGGTGGATTGCATCGAGG
***mir-21* (+1395)**	TGCAACAGACTGGCCTTC	CATGCAAGACTGTTATCCAATCT
mir-30b	GGGAAGGATATAGGAAGGCTGG	GCCCAGGCTAGTCACAAACA
IL-8	GGGCCATCAGTTGCAAATC	GGAAGAAACCACCGGAAGGAA
**Primers for Promoter constructs[a]**		
***mir-125b-1* (-2514 to +106)**	tacgcgtAGAAAGGCCACCAAGATTCAC	tgctagcTGAGAGGAGCGCAACAATGT
***mir-125b-1* (-1129 to +106)**	tacgcgtAAAGGGTCATCTTCCCATCTG	tgctagcTGAGAGGAGCGCAACAATGT
***mir-23b-27b-24-1* (-1396 to+41)**	tacgctGCAGCTAGCAGGGTGATGTT	tctcgagACAGGGAGCGAACAGGTTA
mir-30b	tacgcgtTTAATTCTGGATGCCCTTGCT	tgctagcTTGCCCAGGCTAGTCACAA
***mir-21* (-332 to +1957)**	tacgcgtGAAGTTGGTTTGCCAGTGTTCC	tgctagcTCCCAGAGGTGCCATTTAGC
***mir-21* (+1269 to +1957)**	tacgcgtATCCACCCTCGATGCAATC	tgctagcTCCCAGAGGTGCCATTTAGC
mir-30d	tacgcgtAGACATGAGCCACTGTGCCT	taagcttTGTCTTACAGCTTCCAGTCGG
**Primers for 5'-RACE PCR**		
	**Gene-specific primer (5'-3')**	**Nested gene-specific primer (5'-3')**
mir-30d	GTAGCAGCAAACATCTGACTGAAAGCTTAGCTG	CAGCTTCCAGTCGGGGATGTTTACAAC
mir-125b-1	CTCGCAGCTCCCAAGAGCCTAACC	CGGTAAACATCACAAGTTAGGGTCTCAGGG
mir-30b	GCTGAAGTAAACATCCACCTCCCAGCC	CCCAGCCAATCCATGTATTACAGCTGAG

Listed in this table are all the primers used in this study for the real-time PCR and RACE PCR, as well as those for ChIP analysis and construct generating. [a]Restriction enzyme sites were indicated by lowercase letters.
