# Supplementary material for: NF-kappaB p65-Dependent Transactivation of miRNA Genes following Cryptosporidium parvum Infection Stimulates Epithelial Cell Immune Responses
Source: PLoS Pathog. 2009 Dec 4;5(12):e1000681. doi: 10.1371/journal.ppat.1000681 (PMC2778997; doi:10.1371/journal.ppat.1000681)
Supplement: Table S3 — Prediction of immune-related target genes of C. parvum-responsive miRNAs. Prediction of immune-related target genes for C. parvum-responsive miRNAs was performed with the computerlized predictive algorithms as previously reported [19], [37]–[39]. Some of the predicted targets have been experimentally confirmed [24],[25],[27],[59],[60] and the corresponding miRNAs are in red font. (0.02 MB PDF) [file ppat.1000681.s003.pdf]

Table S3. Prediction of immune-related target genes of *C. parvum*-responsive miRNAs

Pathway	Gene Symbol	miRNA
**AU-Rich Element (ARE) processing machinery**	*KSRP*	miR-23b, miR-27b
	TTP	miR-27b
	TIA1	miR-30b, miR-30c
**Apoptosis signaling**	*p27*	miR-221, miR-222, miR-24
	BCL2	miR-16 [59], miR-15, miR-30, miR-21
	BCL2L1	let-7/miR-98, miR-214
	BCL2L11	miR-24, miR-214, miR-30
	BAK1	miR-125b
	DEDD	miR-16, miR-15, miR-130b
	FASLG	let-7/miR-98, miR-21
	CASP2	miR-125b
	CASP3	let-7/miR-98, miR-30
**Cytokine signaling**	*TLR4*	*let-7i* [24]
	CCL1	miR-21
	CX3CL1	miR-16, miR-15
	IL6	let-7/miR-98
	IL1R1	miR-24
	IL6R	miR-23b
	SOCS4	let-7/miR-98
	CISH	*let-7*/miR-98 [25]
	SOCS1	miR-30b
**Adhesion and B7 costimulatory molecules**	*ICAM1*	miR-221, miR-222 [60]
	CD95	let-7/miR-98, miR-23b, miR-24
	CD40	miR-195, miR-16, miR-15
	B7H1	miR-513 [27], miR-16, miR-15
**Innate pathogen detection**	*IRAK1*	miR-214
	IRAK2	miR-424, miR-16, miR-15
	NALP1	miR-125b

Prediction of immune-related target genes for *C. parvum*-responsive miRNAs was performed with the computerlized predictive algorithms as previously reported [19,37-39]. Some of the predicted targets have been experimentally confirmed and the corresponding miRNAs are in red font.
