# Supplementary figures and images for: NF-kappaB p65-Dependent Transactivation of miRNA Genes following Cryptosporidium parvum Infection Stimulates Epithelial Cell Immune Responses
Source: PLoS Pathog. 2009 Dec 4;5(12):e1000681. doi: 10.1371/journal.ppat.1000681 (PMC2778997; doi:10.1371/journal.ppat.1000681)

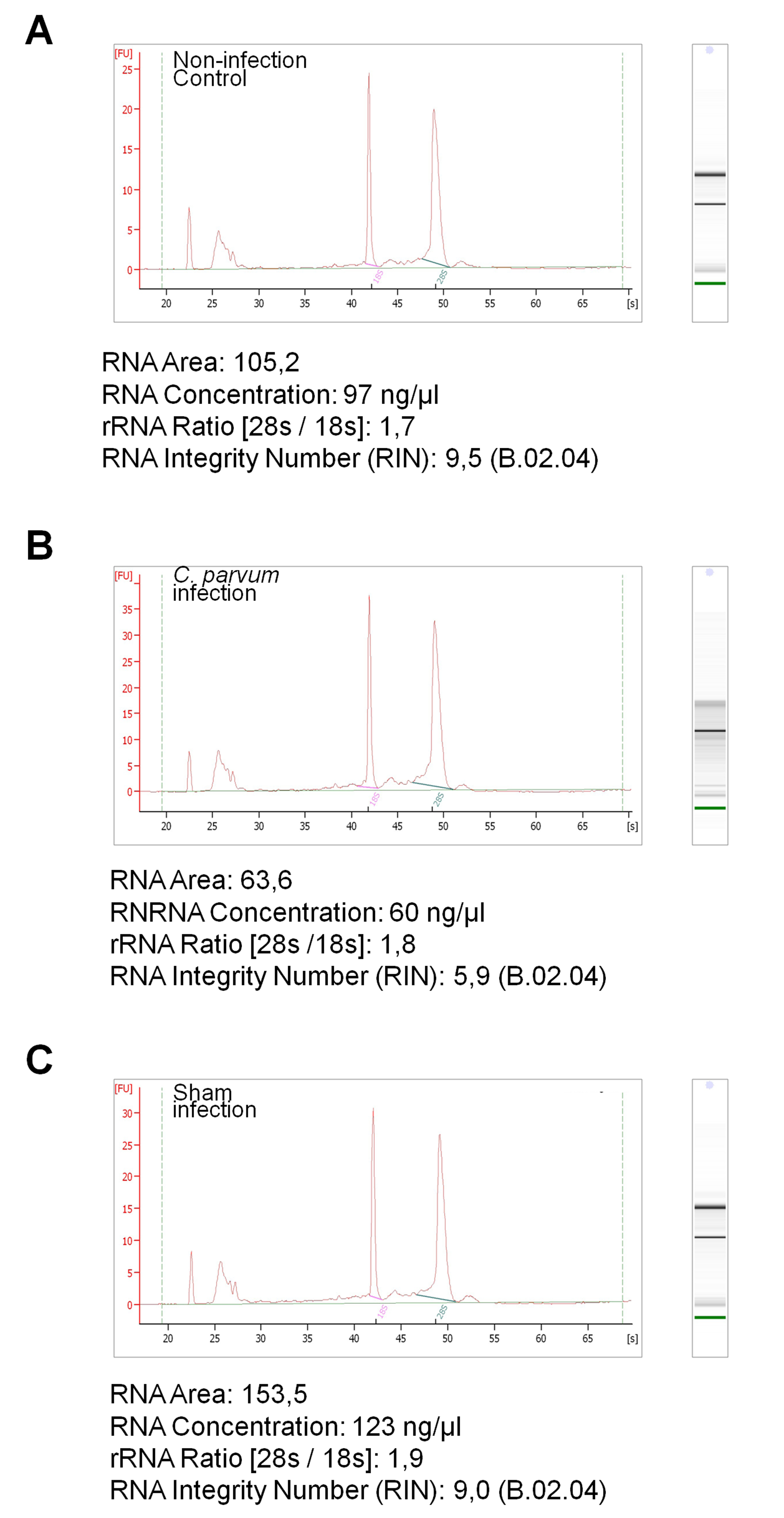

Supplement: Figure S1 — Quality control of RNA. Total RNAs from cells were prepared with the mirVana™ miRNA Isolation Kit according to the manufacturer's instructions (Ambion). The quality of the isolated RNAs was verified by examining the Agilent 2100 Bioanalyzer profile of the sample. Representative RNA profiles from non-infected H69 cells (A), cells exposed to live (B) and heat-inactivated C. parvum oocysts (C) are shown. (1.62 MB TIF) [file ppat.1000681.s004.tif]

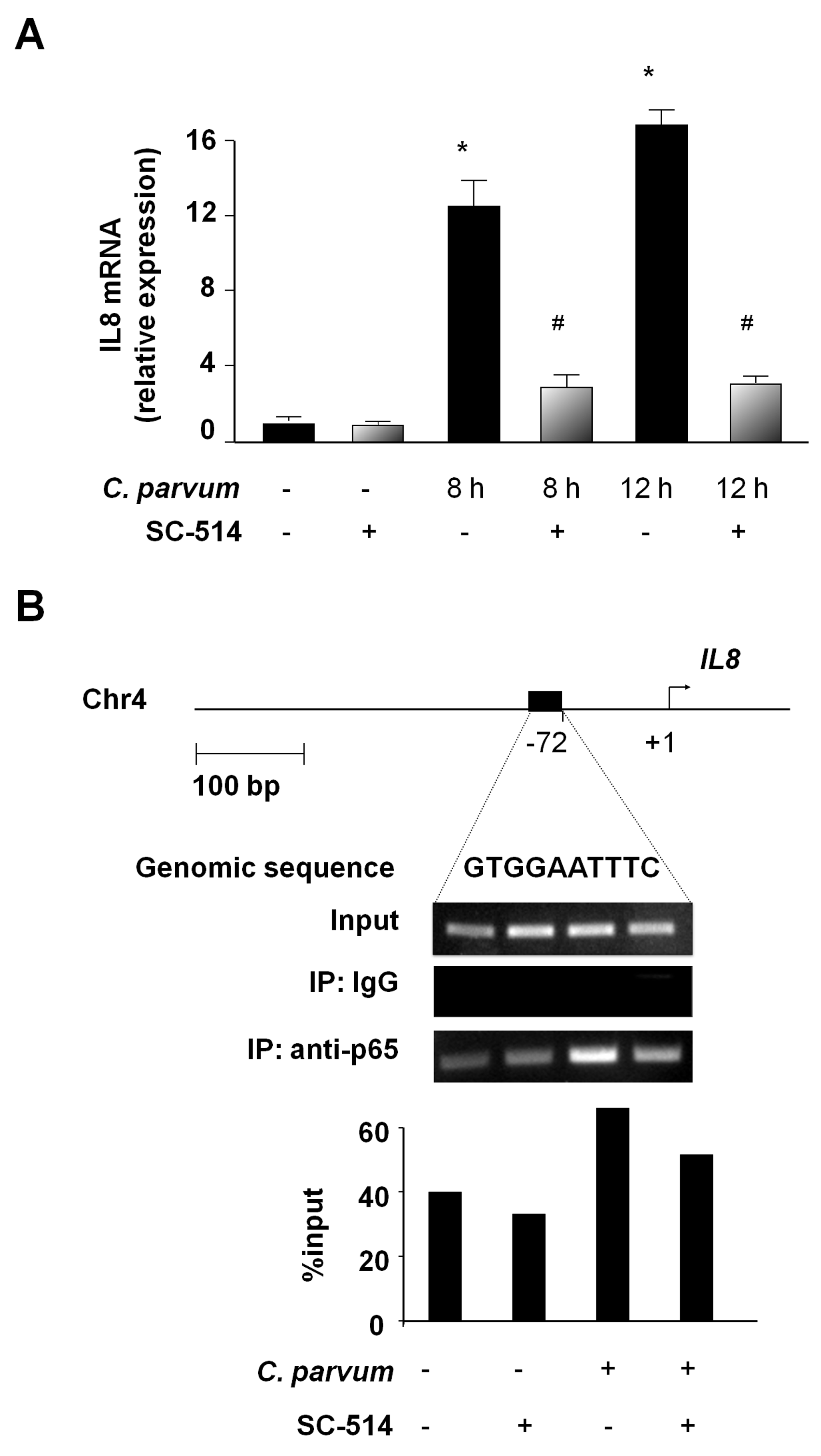

Supplement: Figure S2 — Promoter binding of p65 transactivates the IL-8 gene in cholangiocytes in response to C. parvum infection. (A) p65-dependent upregulation of IL-8 mRNA in cholangiocytes following C. parvum infection. Bars represent the levels of IL-8 mRNA in cells following C. parvum infection in the presence or absence of SC-514 as assessed by real-time PCR. (B) A schematic diagram shows the structure of IL-8 gene. ChIP analysis demonstrated increased binding of p65 to the binding site at IL-8 promoter in cells following infection. *, p<0.05 vs. non-infected cells; #, p<0.05 vs. C. parvum infected cells. (0.28 MB TIF) [file ppat.1000681.s005.tif]

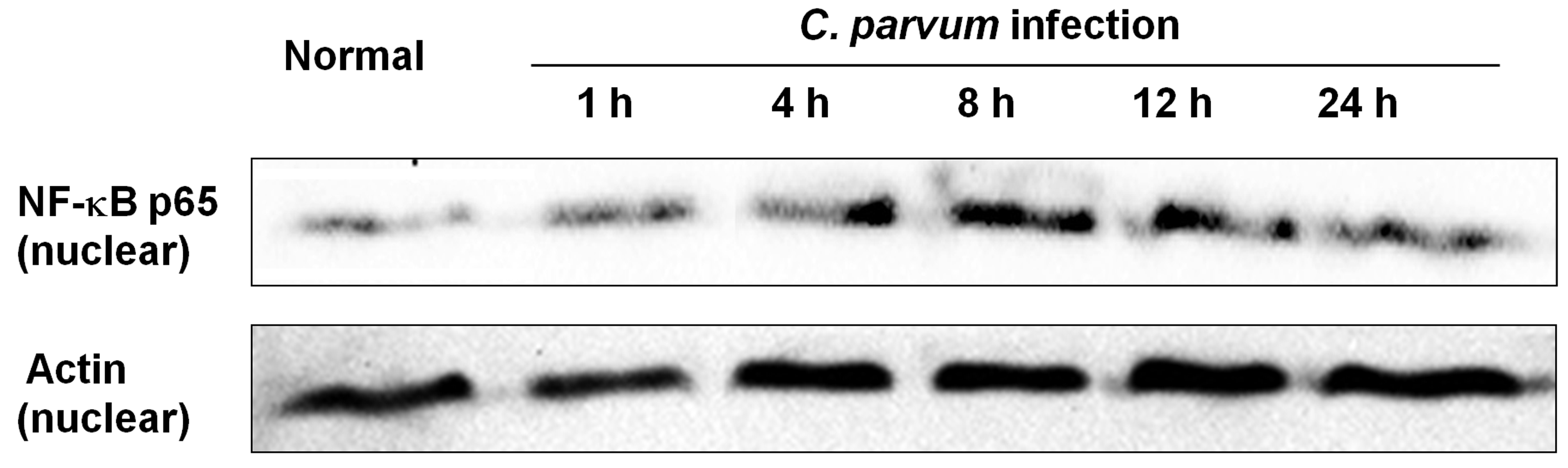

Supplement: Figure S3 — Nuclear translocation of p65 in cholangiocytes cells induced by C. parvum. Cells were exposed to C. parvum for various periods of time and nuclear extracts obtained as described in Protocol S1. The NF-κB p65 subunit was detected by Western blot. Actin was used as a loading control. Representative Western blots are shown. (0.61 MB TIF) [file ppat.1000681.s006.tif]

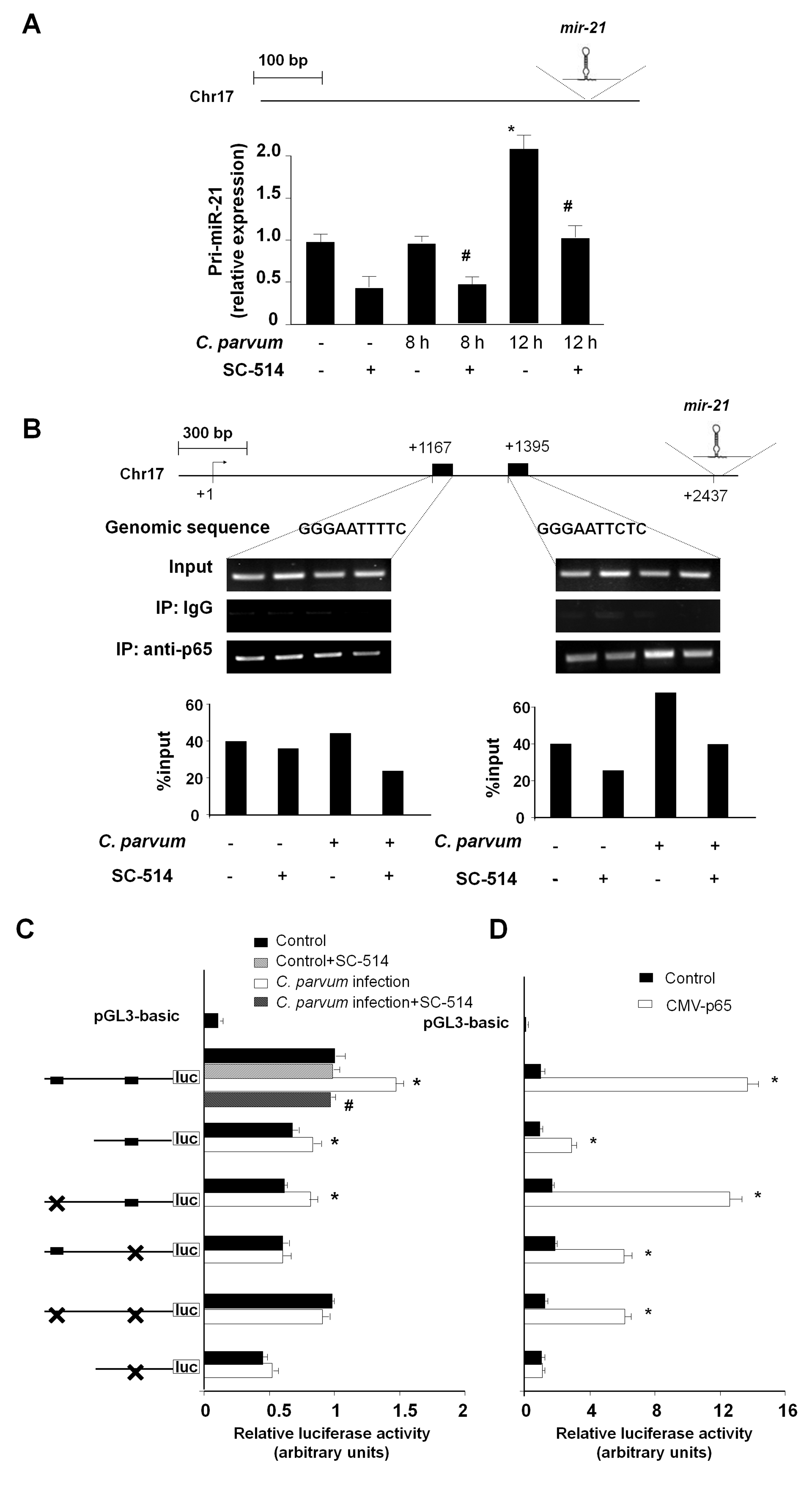

Supplement: Figure S4 — Promoter binding of p65 transactivates mir-21 gene to increase miR-21 expression in biliary epithelial cells in response to C. parvum infection. (A) p65-dependent upregulation of pri-miR-21 in cholangiocytes following C. parvum infection. A schematic diagram illustrates the structure of the mir-21 gene that encodes human miR-21. Bars represent the expression levels of pri-miR-21 in cells following C. parvum infection in the presence or absence of SC-514 as assessed by real-time PCR. (B) C. parvum increases promoter binding of p65 to the mir-21 gene. ChIP analysis revealed increased binding of p65 to the promoter binding site at +1395, but not at +1167 in H69 cells following infection. (C) H69 cells were transfected with various luciferase reporter constructs covering the potential binding sites of the mir-21 promoter and then exposed to C. parvum in the presence or absence of SC-514. A mutant at +1395 blocked C. parvum-induced luciferase reporter activity in transfected cells. (D) H69 cells were co-transfected with the pCMV-p65 to overexpress p65 and the luciferase reporter construct containing the mir-21 promoter. Different from the results in C. parvum-infected cells, a significant increase of luciferase reporter activity was detected in cells co-transfected with the pCMV-p65 and the mutant at +1167. *, p<0.05 vs. the non-infected control (in A and C) or empty pCMV vector control (in D); #, p<0.05 vs. C. parvum infected cells (in A and C). (0.63 MB TIF) [file ppat.1000681.s007.tif]

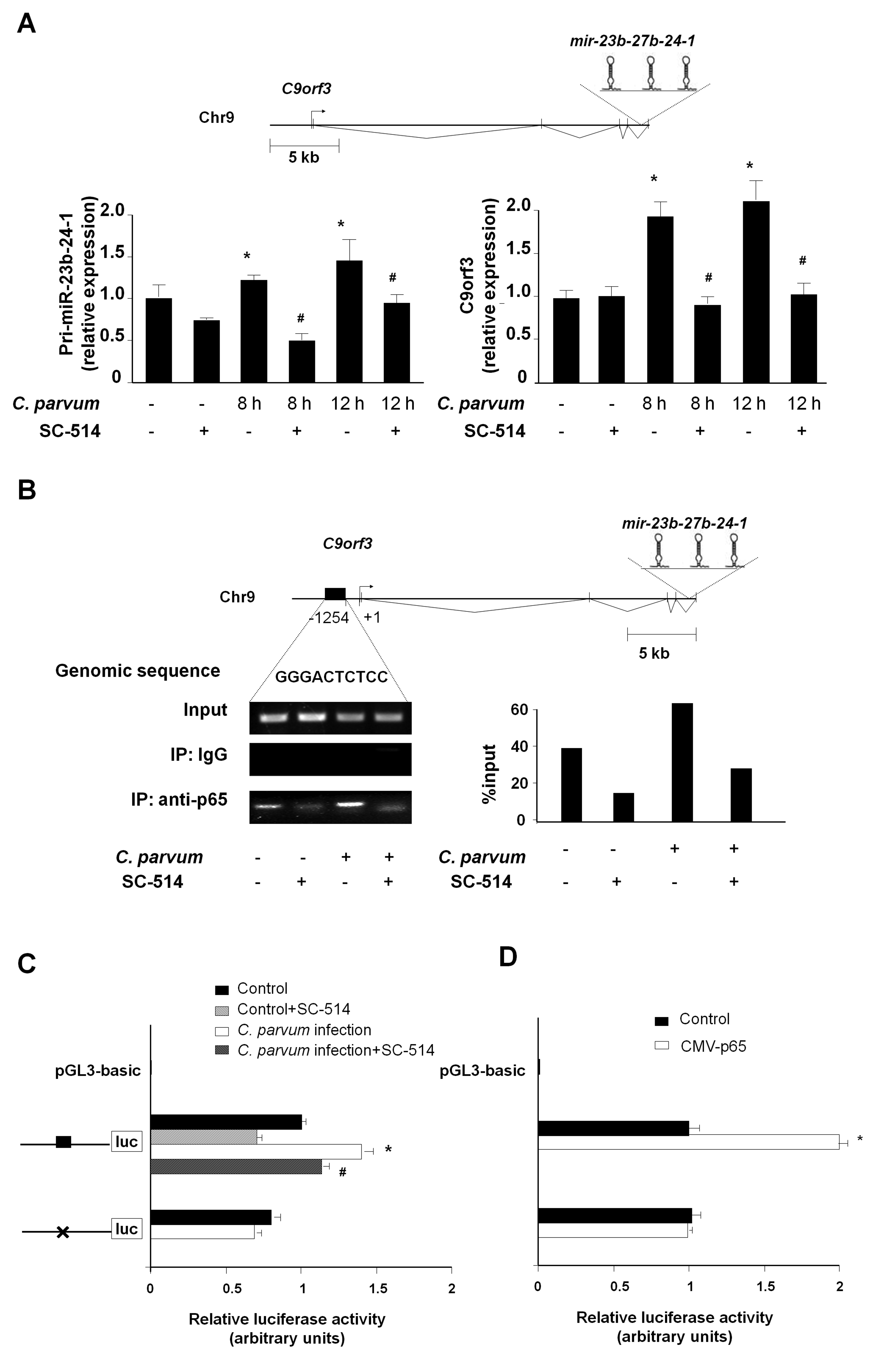

Supplement: Figure S5 — Promoter binding of p65 transactivates the mir-23b-27b-24-1 cluster gene in cholangiocytes in response to C. parvum infection. (A) p65-dependent upregulation of pri-miR-23b-27b-24-1 in cholangiocytes following C. parvum infection. A schematic diagram shows the structure of the mir-23b-27b-24-1 cluster gene. Real-time PCR was used to assess the expression levels of pri-miRNA-23b-27b-24-1 and C9orf3 following C. parvum infection in the presence or absence of SC-514. (B) C. parvum increases promoter binding of p65 to the mir-23b-27b-24-1 cluster gene. The schematic diagram shows one potential NF-κB binding site in the promoter element of mir-23b-27b-24-1. ChIP analysis revealed increased binding of p65 to the binding site at −1254 of the promoter in cells following infection. (C) H69 cells were transfected with luciferase gene reporter constructs with or without mutations in the p65 binding site of the promoter and then exposed to C. parvum in the presence or absence of SC-514. (D) H69 cells were co-transfected with the pCMV-p65 to overexpress p65 and the luciferase reporter gene construct containing the promoter. Cells were then cultured for 24 h followed by measurement of luciferase activity. *, p<0.05 vs. the non-infected control (in A and C) or empty pCMV vector control (in D); #, p<0.05 vs. C. parvum infected cells (in A and C). (0.76 MB TIF) [file ppat.1000681.s008.tif]

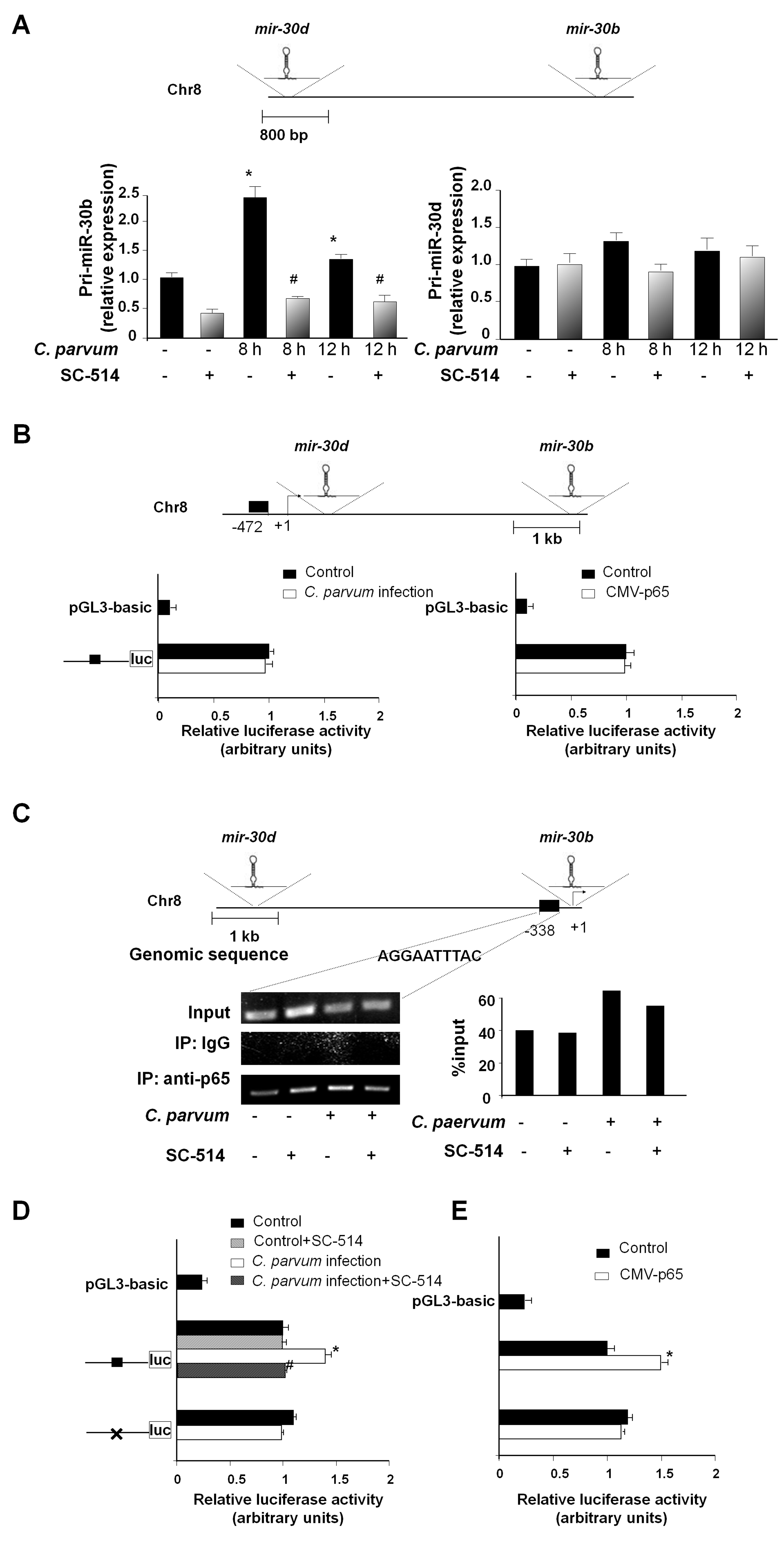

Supplement: Figure S6 — Promoter binding of p65 transactivates the mir-30b gene to increase miR-30b expression in biliary epithelial cells following C. parvum infection. (A) p65-dependent upregulation of pri-miR-30b, but not pri-miR-30d, in cholangiocytes following C. parvum infection. The expression levels of pri-miRNAs in cells following C. parvum infection were assessed by real time PCR in the presence or absence of SC-514. Treatment of cells with SC-514 blocked C. parvum-induced increase of pri-miR-30b, suggesting p65-dependent miR-30b expression. (B) We performed 5′-RACE PCR to identify the 5′end of pri-miR-30d and identified a potential p65 binding site at −472 of its upstream sequence. H69 cells were transfected with the luciferase gene reporter construct covering the potential p65 binding site within the putative promoter of mir-30d and then exposed to C. parvum. These results support that pri-miR-30b and pri-miR-30d are not transcribed from the same gene in human cholangiocytes, inconsistent with previous results suggesting that pri-miR-30b and pri-miR-30d may be transcribed from the same gene on chr8 [31],[40]. (C) To clarify how p65 is involved in the transactivation of miR-30b gene transactivation, we performed 5′-RACE PCR but failed to amplify the corresponding sequence (data not shown). Nevertheless, database analysis revealed one potential binding site for NF-κB in the upstream sequence of miR-30b precursor. ChIP analysis detected an increased binding of p65 to this region in cells following C. parvum infection. (D) Luciferase reporter gene analysis demonstrated a significant increase in luciferase reporter activity in cells following C. parvum infection or overexpressed with p65. *, p<0.05 vs. the non-infected control (in A and D) or empty pCMV vector control (in E); #, p<0.05 vs. C. parvum infected cells (in A and D). (0.73 MB TIF) [file ppat.1000681.s009.tif]

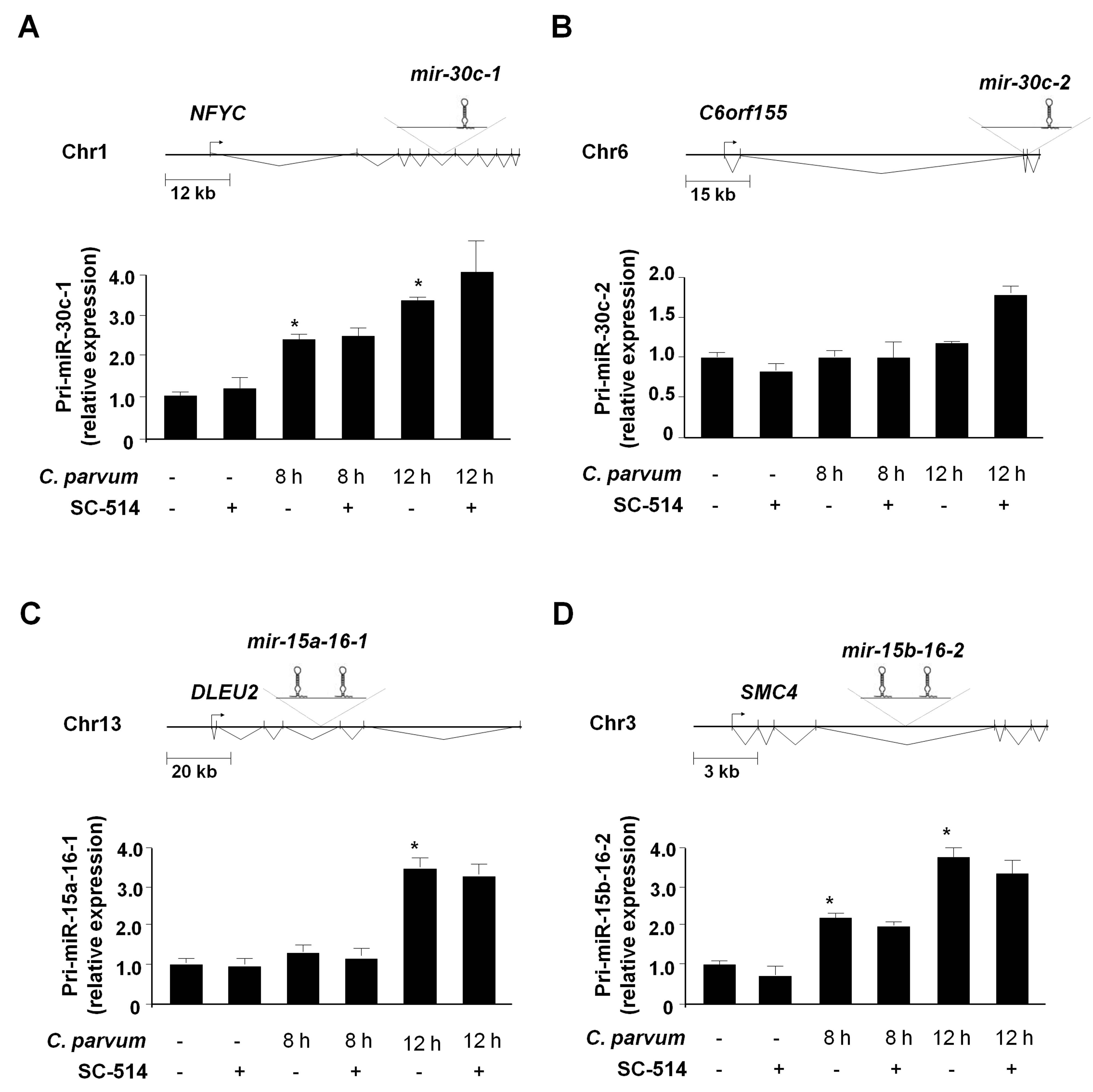

Supplement: Figure S7 — p65-independent expression of miR-30c and miR-16 in cholangiocytes in response to C. parvum infection. (A and B) miR-30c is transcribed from two genes, mir-30c-1 and mir-30c-2, localized on chr1 and chr6, respectively [31]. Real-time PCR analysis revealed an increase of pri-miR-30c-1(A), but not pri-miR-30c-2 (B), in H69 cells following C. parvum infection. Treatment of cells with SC-514 failed to block C. parvum-induced expression of pri-miR-30c-1 (A). (C and D) miR-16 is transcribed from two genes, mir-15a-16-1 and mir-15b-16-2 localized on chr13 and chr3 and clustered with miR-15a and miR-15b, respectively [31]. Increased expression of pri-miR-15a-16-1 (at 12 h; C) and pri-miR-15b-16-2 (at 8 h and 12 h; D) was detected in H69 cells after C. parvum infection. Treatment of cells with SC-514 failed to block either pri-miR-15a-16-1 (C) or pri-miR-15b-16-2 (D). *, p<0.05 vs. the non-infected control. (0.58 MB TIF) [file ppat.1000681.s010.tif]
