# Supplementary material for: NF-kappaB p65-Dependent Transactivation of miRNA Genes following Cryptosporidium parvum Infection Stimulates Epithelial Cell Immune Responses
Source: PLoS Pathog. 2009 Dec 4;5(12):e1000681. doi: 10.1371/journal.ppat.1000681 (PMC2778997; doi:10.1371/journal.ppat.1000681)
Supplement: Protocol S1 — Nuclear translocation of p65. (0.01 MB PDF) [file ppat.1000681.s011.pdf]

Protocol S1

Nuclear translocation of p65. H69 cells were grown to 80% confluence and then infected with *C. parvum* for various times. Cells were trypsinized with trypsin-EDTA, washed with PBS, and cell pellet was resuspended in 1 ml of cold buffer A (10 mM HEPES, 1.5 mM $MgCl_2$, 10 mM KCl, 1 mM DTT). Nuclear pellets were isolated from the whole cell protein by centrifugation at 14,000 rpm for 1 min at 4°C and resuspended in two-thirds packed cell volume of cold buffer B (20 mM HEPES, 1.5 mM $MgCl_2$, 25% glycerol, 420 mM NaCl, 0.2 mM EDTA, 1 mM DTT) with vigorous agitation in the cold room for 30 min. The supernatant containing nuclear proteins was collected after centrifugation at 14,000 rpm for 5 min at 4°C and stored at -70°C. Protein concentration of each nuclear extract was determined and subsequently analyzed by Western blot for p65.
